# Toxicological evaluation of paracetamol and ibuprofen: genetic and hematological alterations in male albino mice

**DOI:** 10.1186/s40360-026-01111-5

**Published:** 2026-03-28

**Authors:** Shimaa M. Elgingihy, Ayaat M. Elmaghraby, Yehia A. Mustafa, Mohamed A. Elseehy, Amany S. Haggag, Salah M. Abdel-Rahman

**Affiliations:** 1 Department of Genetics, Faculty of Agriculture, Alexandria University, Alexandria, Egypt; 2 Department of Nucleic Acid Research, Genetic Engineering and Biotechnology Research Institute (GEBRI), City of Scientific Research and Technological Applications (SRTA-City), Alexandria, Egypt

**Keywords:** Paracetamol, Ibuprofen, Albino mice, Drug-induced toxicity, Hematology, Histopathology, TNF-α, Connexin 43, Gene expression

## Abstract

Antipyretic drugs are widely used to manage fever and pain and are generally regarded as safe when administered within recommended therapeutic ranges. Nevertheless, concerns remain regarding the potential biological effects associated with repeated or sub-chronic exposure. The present study evaluated the hematological, histopathological, and molecular effects of two commonly used antipyretics, paracetamol and ibuprofen, following repeated administration in male albino mice. Hematological analysis revealed drug-related alterations in selected blood parameters, with more pronounced changes observed in ibuprofen-treated groups, while paracetamol exposure was associated with comparatively milder effects. Qualitative histopathological examination demonstrated organ-specific structural alterations in the testes, liver, kidneys, and stomach, which were generally mild in nature and more frequently observed at higher experimental dose levels. No evidence of overt testicular failure or severe tissue damage was detected within the exposure period. At the molecular level, repeated exposure to paracetamol and ibuprofen was associated with downregulation of TNF-α and connexin 43 mRNA expression in peripheral blood, suggesting early systemic transcriptional responses accompanying the observed histological changes. However, molecular findings were limited to mRNA expression analysis and did not include protein-level or tissue-specific validation. Overall, the results indicate that repeated sub-chronic exposure to paracetamol and ibuprofen at therapeutic-equivalent dose ranges may be associated with early hematological, histological, and molecular alterations in mice. These findings highlight the importance of cautious and rational use of widely prescribed antipyretic drugs and underscore the need for further studies to elucidate long-term safety, underlying mechanisms, and tissue-specific functional outcomes.

## Introduction

Acetaminophen, sometimes known as paracetamol, is the most commonly used over-the-counter (OTC) medication worldwide. Its exact mode of action is yet unknown. At least a few metabolic pathways are thought to be involved in the analgesic and antipyretic effects of paracetamol, which is now thought to be a multidirectional medication. Paracetamol exerts its analgesic and antipyretic effects partly via cyclooxygenase inhibition and modulation of serotonergic and endocannabinoid pathways [1]. According to data from COX-1 transgenic mice, paracetamol produces its analgesic and particularly thermoregulatory effects (antipyresis and hypothermia) by blocking an enzyme that is unique to the COX-1 type. The COX-1 and COX-2 enzymes, including PGE2 (prostaglandin E2), are inhibited by paracetamol. The transmission of nociceptive pain at the location of pain start is significantly influenced by the chemical makeup of paracetamol [2]. Paracetamol-induced toxicity is mainly mediated by oxidative stress, inflammation, and apoptosis [3]. At therapeutic doses, paracetamol is generally considered safe; however, overdose or prolonged use has been associated with toxic effects, primarily affecting the liver and, to a lesser extent, the kidneys [4]. The most common cause of acute liver failure (ALF) and a key reason for liver transplantation in Western nations is paracetamol overdose. At therapeutic levels, the majority of paracetamol is conjugated and eliminated; however, a tiny portion is converted into the reactive metabolite N-acetyl-p-benzoquinone imine (NAPQI) by Cyp2E1 and Cyp1A2. Overdosing on NAPQI causes oxidative stress, hepatocellular apoptosis, and mitochondrial malfunction by depleting glutathione (GSH) and binding to cellular proteins [5].

N-acetylcysteine reduced acute kidney damage induced by a therapeutic dose of paracetamol for 15 days, most likely by altering kidney injury markers in renal tissues, such as KIM-1 (Kidney Injury Molecule-1) and CHOP/GADD153 [6]. Even in the initial hours of intoxication, paracetamol caused biochemical changes, and within 12 h, all biochemical, histological, inflammatory, and redox status parameters had changed. Thus, we emphasize the significance of early detection and treatment of APAP intoxication. To restore important functions during advanced phases of intoxication, studies including drugs that stop the progression of liver lesions are required [7]. Early paracetamol treatment’s effects on testosterone levels in the testicles and the expression of genes crucial for steroid biosynthesis and reproduction in the progeny of male rats. Testosterone levels were measured in rats given varying dosages of paracetamol over an extended period of time. The findings indicated that paracetamol lowers testicular testosterone levels and leads in compensatory transactivation of genes crucial for steroidogenesis and reproduction. Significant overexpression of a number of genes related to steroid production and cholesterol transport was used to demonstrate this. One potential mechanism that preserves and stops the loss of steroidogenic function could be the upregulation of these genes, along with a concurrent decrease in testosterone in the testicles [8]. More care should be used when using paracetamol during pregnancy since some findings indicate that it may cause the placenta to react in an immune-inflammatory manner [9].

Ibuprofen is a drug used to treat and manage mild to severe discomfort, rheumatoid arthritis, fever, dysmenorrhea, and osteoarthritis. It is also available as an over-the-counter pain reliever, typically in the moderate range. It belongs to the group of medications called nonsteroidal anti-inflammatory drugs (NSAIDs) [10].

Ibuprofen is a nonsteroidal anti-inflammatory drug that lowers the production of prostaglandin fatty acids by blocking the cyclooxygenase (COX) enzyme. Like other nonsteroidal anti-inflammatory drugs, ibuprofen inhibits platelet aggregation, but, because of its low effectiveness and reversibility, it is not used to treat this condition [11]. Ibuprofen is commonly associated with gastrointestinal irritation, while severe gastrointestinal complications are less frequently observed, particularly at recommended therapeutic doses [12]. Numerous genes linked to the control of inflammation and cell migration showed elevated expression when taking ibuprofen [13].

Exposure to non-steroidal anti-inflammatory drugs (NSAIDs) has been shown to alter the gonadal transcriptome [14], leading to disruptions in testicular and ovarian physiology that may subsequently affect the timing of puberty, a sensitive developmental endpoint [15].

showed many of the changes in theca-interstitial cells brought on by inflammatory stimuli are reversed when ibuprofen is added [16].

TNF-α is a pleiotropic pro-inflammatory cytokine mainly produced by monocytes and macrophages. Through a variety of mechanisms, TNF-α can cause inflammation, proliferation, and apoptosis. The development of cirrhosis from chronic hepatitis has been associated with TNFα [17].

The TNF-α ligand interacts with two receptors to start signal transduction processes that activate pathways controlling cellular survival, differentiation, and proliferation [18]. A gap junction protein called Connexin 43 (Cx43) is vital for intercellular communication and plays a significant part in tissue homeostasis and the development of illness [19]. Inflammatory mediators including TNF-α, which can increase macrophage production of Cx43 and affect apoptosis, modify its expression [20].

TNF-α influences inflammatory reactions and apoptosis via controlling Cx43 expression and signaling [21, 22]. Connexin expression is modulated across tissues by cytokines including TNF-α and interleukins, creating feedback loops that regulate inflammation [23]. Drug-induced organ toxicity, where oxidative stress and inflammation lead to cell death and tissue malfunction in the liver, kidney, and gastrointestinal tract, has been linked to Cx43 dysregulation [24] .

Despite extensive literature on the toxicological effects of paracetamol and ibuprofen, limited studies have combined hematological, histopathological, and blood-based molecular endpoints within a single sub-chronic exposure model. So, the present study aims to provide an integrated assessment of early systemic responses following repeated exposure to these commonly used analgesics, with a comparative evaluation between paracetamol and ibuprofen.

### Sub-chronic toxicological effects of paracetamol and ibuprofen

Repeated and prolonged administration of paracetamol and ibuprofen has been widely reported to induce cumulative toxic effects in rodent models. Chronic paracetamol exposure in Wistar rats caused pronounced hepatocellular damage, oxidative stress imbalance, and increased DNA fragmentation, reflecting dose- and duration-dependent systemic toxicity [25]. Likewise, subchronic ibuprofen treatment in rats resulted in significant elevations in liver and kidney injury biomarkers, including transaminases, urea, and creatinine, accompanied by hematological disturbances indicative of impaired physiological homeostasis [26]. Recent toxicological assessments further demonstrate that prolonged ibuprofen exposure amplifies oxidative and cellular stress responses, reinforcing concerns regarding cumulative NSAID-induced toxicity beyond their intended analgesic effects [27].

Repeated administration of commonly used antipyretic and analgesic drugs such as paracetamol and ibuprofen has been associated with measurable toxicological effects in experimental animal models. In Swiss albino mice, one-month repeated exposure to paracetamol and ibuprofen resulted in significant alterations in immunological and biochemical parameters, indicating that sub-chronic exposure may induce systemic toxicity beyond acute effects [28].

In addition to biochemical and histological alterations, repeated exposure to non-steroidal anti-inflammatory drugs has been linked to genetic damage. Ibuprofen administration was reported to induce genotoxic effects in mouse bone marrow cells in vivo, suggesting that hematological and genetic alterations may occur following repeated dosing [29]. Moreover, temporal analysis of paracetamol-induced hepatotoxicity demonstrated progressive molecular and histopathological liver damage over time, supporting the concept that repeated exposure contributes to cumulative hepatic stress and injury [30].

## Materials and methods

### Ethics

All animal procedures were reviewed and approved by the Institutional Animal Care and Use Committee (IACUC), Faculty of Medicine, Alexandria University (AlexU-IACUC) (Approval No: AU-08-22-08-10-1-97). Humane anesthesia and euthanasia protocols were applied, and all efforts were made to minimize animal suffering. All experiments were conducted in accordance with institutional guidelines and the ARRIVE 2.0 recommendations.

### Animal handling, anesthesia, and euthanasia

Animals were randomly allocated to experimental groups to minimize selection bias. Histopathological and hematological assessments were performed in a blinded manner by an independent pathologist/investigator. Mice were housed under standard laboratory conditions with controlled temperature and humidity, a 12 h light/dark cycle, free access to food and water, and environmental enrichment.

Deep anesthesia was induced using amobarbital sodium administered intraperitoneally at a dose of 150–200 mg/kg, in accordance with the approved IACUC protocol and the AVMA Guidelines for the Euthanasia of Animals (AVMA, 2020) [31]. Adequate anesthetic depth was confirmed by the absence of pedal withdrawal and corneal reflexes. Following confirmation of deep anesthesia, animals were humanely euthanized by cervical dislocation to ensure rapid and painless death.

### Experimental animal design

From the animal house at Pharos University’s Faculty of Pharmacy in Alexandria, Egypt, forty male albino mice were chosen at random. The 40 male albino mice (Mus musculus, weighing 25–30 g) were moved to Alexandria University’s Medical Research Institute and placed there. The animals were housed in plastic cages, three per cage, under controlled environmental conditions (temperature: 25 °C, humidity: 50%, and a 12-hour light–dark cycle). The animals were then allowed unrestricted access to food and water. Before the trial started, the animals were acclimated for two weeks and watched every day for any unusual symptoms.

Animals were randomly assigned to experimental groups and received paracetamol or ibuprofen at predetermined doses (mg/kg body weight) via oral gavage once daily for the duration of the experimental period. Dose selection was based on previously published rodent toxicology studies and human-equivalent dose (HED) conversion using body surface area normalization, according to FDA guidelines. The HED was calculated using the following equation: HED (mg/kg) = Animal dose (mg/kg) × (Km animal / Km human). Based on this approach, the selected doses represent sub-chronic therapeutic exposure ranges relevant to human use.

### Experimental animal groups

Following acclimatization, forty male albino mice were randomly assigned to one control group (C; *n* = 5) and two experimental groups: a paracetamol-treated group (A; *n* = 15) and an ibuprofen-treated group (B; *n* = 20). Each experimental group was further subdivided into dose-specific subgroups (*n* = 5 per subgroup), as shown in Table 1.

Human-equivalent doses (HEDs) were calculated using body surface area normalization according to U.S. Food and Drug Administration (FDA) guidelines using the following equation:

$$\eqalign{ HED{\rm{ }}\left( {mg/kg} \right){\rm{ }} = & Animal{\rm{ }}dose{\rm{ }}\left( {mg/kg} \right){\rm{ }} \times {\rm{ }} \cr & \left( {Km\_mouse{\rm{ }}/{\rm{ }}Km\_human} \right), \cr} $$

where Km values are 3 for mice and 37 for humans.

Group 1: Vehicle control (sterile distilled water, oral gavage, 10 ml/kg), Paracetamol and ibuprofen were administered once daily by oral gavage (10 mL/kg body weight) at a fixed time each day to ensure consistent dosing, using distilled water as the vehicle. Commercial tablet formulations of both drugs were finely ground using a sterilized mortar and pestle and dissolved in distilled water with continuous shaking until complete solubilization was achieved. A corresponding vehicle control group received sterile distilled water via oral gavage at the same volume and schedule.

Although commercial tablet formulations reflect real-world drug exposure, the presence of inactive excipients may influence dissolution, absorption, or bioavailability. This is acknowledged as a methodological limitation.

The administered doses listed in Table 1 refer exclusively to mouse doses. Human-equivalent doses (HEDs) were calculated separately using body surface area normalization and are presented in Table 2 for comparative interpretation only.

Human-equivalent doses (HEDs) were calculated using body surface area normalization according to FDA guidelines and are provided for comparative context only. These values are not intended to imply direct therapeutic equivalence or clinical relevance in humans. The selected dose ranges were based on previously published rodent toxicology studies and were designed to model repeated sub-chronic exposure in mice rather than human therapeutic or overdose conditions.

The selected dose ranges were based on previously published rodent studies and were intended to model sub-chronic exposure rather than therapeutic dosing in humans, and were chosen to induce early systemic, hematological, and histological responses without modeling acute overdose. Given the higher metabolic rate of rodents, repeated daily administration over a 30-day period was employed to assess sub-chronic exposure effects.

Commercial tablet formulations were used rather than pure active pharmaceutical ingredients. Although this approach reflects real-world drug exposure, the presence of excipients may introduce variability in absorption and bioavailability. This is acknowledged as a limitation of the study.

**Table 1:** Distribution of experimental groups and subgroups of mice

Group	Subgroup	*n*	Drug	Dose (mg/kg/day, mice)
C	–	5	None	–
A	A1	5	Paracetamol	12.5
A	A2	5	Paracetamol	25
A	A3	5	Paracetamol	50
B	B1	5	Ibuprofen	10
B	B2	5	Ibuprofen	20
B	B3	5	Ibuprofen	30
B	B4	5	Ibuprofen	50

**Table 2:** Conversion of mice doses to human-equivalent doses (HEDs)

Drug	Mice dose (mg/kg)	HED (mg/kg)	Approximate human dose (mg/day, 70 kg)
Paracetamol	12.5	1.01	~ 71
Paracetamol	25	2.03	~ 142
Paracetamol	50	4.05	~ 284
Ibuprofen	10	0.81	~ 57
Ibuprofen	20	1.62	~ 113
Ibuprofen	30	2.43	~ 170
Ibuprofen	50	4.05	~ 284

### Collection and preparation of samples for biological techniques

At the end of the experimental period, mice were fasted for 24 h prior to sample collection to minimize metabolic variability. Blood samples were collected via cardiac puncture under deep anesthesia and immediately transferred into EDTA-coated tubes. Complete blood count (CBC) analysis was performed immediately on fresh whole blood samples. CBC samples were not frozen or stored prior to analysis.

Following CBC analysis, the remaining blood was centrifuged to separate plasma, which was collected and stored for subsequent biochemical analyses. The cellular fraction was processed immediately for RNA extraction and gene expression analysis.

All animal procedures were conducted in compliance with the Institutional Animal Ethics Committee (IAEC) and in accordance with the American Veterinary Medical Association (AVMA) Guidelines for the Euthanasia of Animals (2020). Animals were anesthetized and euthanized using an intraperitoneal injection of amobarbital (150–200 mg/kg), as approved by the Institutional Animal Care and Use Committee (IACUC).

Following euthanasia, the liver, kidneys, stomach, and testes were rapidly excised and rinsed in ice-cold saline. Each tissue was divided into two portions: one portion was snap-frozen and stored at − 80 °C for molecular analyses, and the other was fixed in 10% neutral buffered formalin for histopathological examination.

Total RNA was extracted immediately from peripheral blood samples using a commercially available RNA extraction kit, following the manufacturer’s protocol. RNA concentration and purity were assessed spectrophotometrically by measuring absorbance at 260 and 280 nm. Complementary DNA (cDNA) was synthesized from equal amounts of total RNA using a reverse transcription kit. Quantitative real-time PCR (qRT-PCR) was subsequently performed using SYBR Green chemistry on a real-time PCR system to quantify the relative expression of TNF-α and connexin 43. Gene expression levels were normalized to GAPDH as an internal reference gene and calculated using the 2⁻ΔΔCt method.

### Hematological analysis

Complete blood count (CBC), including red blood cells (RBCs), white blood cells (WBCs), platelets (PLT), lymphocytes, and hemoglobin (HGB), was performed on fresh EDTA-anticoagulated whole blood immediately after collection using an automated hematology analyzer (Sysmex XN-1000, Sysmex Corporation, Kobe, Japan). CBC analysis was performed within 1 h of blood collection. Blood samples were anonymized using coded identifiers prior to analysis.

The Sysmex XN-1000 is a fully automated hematology analyzer that operates based on impedance and fluorescent flow cytometry principles, allowing accurate quantitative and qualitative assessment of blood cell populations. The analyzer utilizes advanced optical and electronic detection systems to ensure high analytical precision and reproducibility of hematological parameters. All analyses were conducted according to the manufacturer’s instructions in the Pathology Department, Faculty of Medicine, Alexandria University. All samples were analyzed on the same day of collection to avoid storage-related artifacts.

Hematological parameters were reported using standard units (×10³/µL for WBCs, ×10⁶/µL for RBCs, g/dL for hemoglobin, and ×10³/µL for platelets), and reference ranges for mice were provided by the analyzer manufacturer.

Following completion of CBC analysis, the remaining blood samples were centrifuged at 2000 × g for 10 min to separate plasma, which was subsequently stored at − 80 °C for future biochemical analyses, when required.

### Histopathological studies

All animal groups C (control), A (A1, A2, and A3), and B (B1, B2, B3, and B4) had their liver, kidney, testes, and stomach tissues sliced into slices and promptly preserved in 10% neutral buffered formalin for a full day. Subsequently, they were cleansed under running water, dehydrated in progressively higher alcohol grades (70, 80, 95, and pure alcohol), and cleaned by submerging them in xylene before being impregnated in molten paraffin wax for an hour at 60 °C. After being embedded in paraffin, the samples were allowed to harden at room temperature. Sections of the prior tissues that were 5 μm thick were cut with a rotary microtome and placed on sterile glass slides. To detect any histological alterations in these tissues, these slices were stained using a standard hematoxylin and eosin (H&E) mixture and examined under a light microscope. This section was completed at Alexandria University in Egypt’s Faculty of Medicine, Histopathology Department.

Histological evaluation was performed on coded slides, and the examiner was blinded to the experimental groups.

### SYBR Green–based quantitative real-time polymerase chain reaction (qRT-PCR)

Total RNA was isolated from peripheral blood samples collected from male albino mice in the control and treated groups using the Gene JET RNA Purification Kit (Geneaid Biotech, Korea), following 30 days of daily oral administration of paracetamol or ibuprofen. RNA concentration and purity were assessed using a NanoDrop spectrophotometer (A260/A280 and A260/A230), and all samples were normalized to a uniform concentration prior to analysis. Gene expression of tumor necrosis factor alpha (TNF-α) and connexin 43 (Cx43) was quantified using SYBR Green–based one-step qRT-PCR on a Bio-Rad real-time PCR system. Glyceraldehyde-3-phosphate dehydrogenase (GAPDH) was used as the reference gene for normalization. Primer sequences and expected amplicon sizes are provided in Table 3. Reverse transcription was performed at 60 °C for 30 min, followed by initial denaturation at 95 °C for 10 min. PCR amplification was carried out for 40 cycles, each consisting of denaturation at 95 °C for 5 s and annealing/extension at 60 °C for 30 s. Each sample was analyzed in technical triplicates, with *n* = 5 biological replicates per group. Amplification specificity was confirmed by melt curve analysis, and no-template controls (NTC) as well as no–reverse transcription (no-RT) controls were included in each run to exclude contamination and genomic DNA amplification. Relative mRNA expression levels were calculated using the comparative Ct (2⁻ΔΔCt) method after normalization to GAPDH. Peripheral blood RNA was used to assess TNF-α and Cx43 expression, providing a systemic overview of transcriptional changes; however, this approach primarily reflects leukocyte-derived expression and may not represent tissue-specific gene regulation in organs such as the liver, kidney, stomach, or testes, which is acknowledged as a limitation of the study.

**Table 3:** Primer sequences used for SYBR Green–based qRT-PCR

Gene	Primer sequence (5′–3′)	Annealing temperature (°C)	Accession number	Reference
**TNF-α**	**F**: CTGGCAGCTCTTCTCAAAGC **R**: CCAGGTCATAGAGAGGCTCAA	61	**AB039231.1**	(Botrous et al. 2024) [32]
**Connexin 43 (Cx43)**	**F**: CAGAACAAGTCTGCACGA **R**: TCTAACTTGGAGCGCAGA	60	**AY555737.1**	(Shkedif, & Abdelmonsif, 2020) [33]
**GAPDH**	**F**: CTGGCCAAGGTCATCCA **R**: CCAGCATCAAAGGTGGAA	60	**XM_063285518.1**	(Botrous et al. 2025) [34]

### Statistical analysis

Statistical analysis was performed using SPSS software (Version 13.0; SPSS Inc., Chicago, IL, USA). Data are presented as mean ± standard deviation (SD). Prior to statistical comparisons, data distribution was assessed for normality using the Shapiro–Wilk test, and homogeneity of variances was evaluated using Levene’s test.

For comparisons among multiple experimental groups, one-way analysis of variance (ANOVA) was applied for normally distributed data, followed by Tukey’s post hoc test to correct for multiple comparisons. When data did not meet the assumptions of normality, the non-parametric Kruskal–Wallis test was used, followed by Dunn’s post hoc test.

An a priori sample size calculation was performed using G*Power software. Assuming a medium-to-large effect size (f = 0.40) based on previous toxicological studies, a significance level of α = 0.05, and a statistical power (1–β) of 0.80, the minimum required sample size was estimated to be 5 animals per subgroup.

A significance level of *P* < 0.05 was considered statistically significant, and exact P-values are reported where applicable. The sample size for each experimental subgroup was *n* = 5 animals.

All samples were coded prior to analysis, and Histopathological evaluation was performed using coded samples, such that the examiner was blinded to group allocation. Statistical analysis was performed by a supervisor who was aware of the experimental groups. until completion of data analysis.

Data were analyzed using SPSS v13. Normality was checked, and comparisons between two groups were performed using Student’s t-test, with p-values reported for all comparisons. Differences were considered significant at *p* < 0.05.

### Randomization and blinding

Animals were randomly assigned to experimental groups following acclimatization. Sample collection and labeling were performed by the authors. Histopathological evaluation was conducted using coded slides, and the examiner was blinded to group allocation during microscopic assessment. Statistical analysis was not blinded, which is acknowledged as a limitation.

## Results

### Body weight

In the present study, the body weight measurements were performed for all groups of 40 male mice. Where the body weight of mice was measured for all groups before and after treatment with paracetamol and ibuprofen, as follows:

- In group C (control), the average body weight of mice increased by 10 g after a whole month.
- In the paracetamol-treated groups (Group A), body weight increased over the experimental period in all subgroups; however, the magnitude of weight gain was lower than that observed in the control group, with the smallest increase recorded in group A3. The mean body weight gain after 30 days was 7.9 g in A1, 5.9 g in A2, and 2.7 g in A3. These differences did not reach statistical significance.
- In the ibuprofen-treated groups (Group B), body weight increased over the experimental period in all subgroups; however, the magnitude of weight gain was lower than that of the control group, with the smallest increase observed in the high-dose group (B4). The mean body weight gain after 30 days was 6.7 g in B1, 4.8 g in B2, 3.8 g in B3, and 2.8 g in B4. These differences did not reach statistical significance (*p* > 0.05).

### Hematological analysis

After CBC (Complete Blood Count) analysis for blood samples collected from all mice groups, the following result was observed, as shown in Table 4:

Data are expressed as Mean ± SD.

**Table 4:** Hematological analysis of blood samples of the tested mice groups

Test	Groups	C	A1	A2	A3	B1	B2	B3	B4
**WBCs** ×10³/µL	M ± SD Significance	10.8 ± 0.3	9.21 ± 0.003	8.12 ± 0.01	8.54 ± 0.02	5.52*± 0.01	5.23^*^± 0.06	4.57*± 0.07	4.1^*^± 0.04
**RBCs** ×10⁶/µL	M ± SD Significance	6.5 ± 0.2	7.32^*^±0.06	7.26^*^±0.03	7.26^*^±0.04	8.08 ± 0.04	8.17^*^± 0.04	8.31^*^± 0.18	6.72 ± 0.05
**HGB** g/dL	M ± SD Significance	15.8 ± 0.3	15.8 ± 0.25	10.1^*^±0.06	10.3^*^±0.06	12.3 ± 0.05	12.5 ± 0.18	13.3 ± 0.10	9.4^*^±0.09
**PLT** ×10³/µL	M ± SD Significance	1219 ± 26	827 ± 0.58	720 ± 0.61	683^*^±1.20	1111 ± 1.53	760 ± 2.65	754 ± 3.21	417^*^±1.15
**LYMPH** ×10³/µL	M ± SD Significance	8.0 ± 0.2	7.47 ± 0.04	6.99 ± 0.11	4.75^*^±0.01	4.04 ± 0.07	3.52^*^±0.03	2.01^*^±0.06	1.91^*^±0.03

Values are expressed as the mean ± SD from independent biological replicates (*n* = 5 per group) of the experiment. Statistical significance was determined by one-way ANOVA followed by LSD post-hoc test, Significant differences were analyzed using one-way ANOVA, where *P < 0.05 vs. the negative control group (C)

#### White blood cells (WBCs)

- In group C (control), the white blood cell count was within the normal physiological range.
- In the paracetamol-treated groups (Group A), white blood cell counts showed a non-significant decrease relative to the control group (*p* > 0.05, one-way ANOVA followed by LSD post-hoc test).
- In the ibuprofen-treated groups (Group B), white blood cell counts showed a statistically significant decrease compared with the control group, with a dose-dependent reduction observed across B1–B4 (*p* < 0.05, one-way ANOVA followed by LSD post-hoc test).
- Overall, repeated exposure to both paracetamol and ibuprofen was associated with a reduction in circulating WBC counts; statistical significance was observed only in the ibuprofen-treated groups.

#### Red blood cells (RBCs)

- In group C (control), the red blood cell count was within the normal range.
- In the paracetamol-treated groups (Group A), RBC counts showed a statistically significant increase in the moderate- and high-dose groups (A2 and A3) compared with the control group (*p* < 0.05).
- In the ibuprofen-treated groups (Group B), RBC counts showed a significant elevation in groups B2 and B3, while no significant changes were observed in B1 and B4 (*p* < 0.05, one-way ANOVA followed by LSD post-hoc test).

#### Hemoglobin (HGB)

- In group C (control), hemoglobin levels were within the normal range.
- In the paracetamol-treated groups (Group A), hemoglobin levels showed a statistically significant decrease in the moderate- and high-dose groups (A2 and A3) compared with the control group (*p* < 0.05, one-way ANOVA followed by LSD post-hoc test).
- In the ibuprofen-treated groups (Group B), a significant reduction in hemoglobin concentration was observed in groups B3 and B4, while lower-dose groups did not show significant changes (*p* < 0.05).

#### Platelets (PLT)

- In group C (control), platelet counts were within the normal physiological range.
- In the paracetamol-treated groups (Group A), platelet counts showed a decreasing trend compared with the control group, with the lowest mean value observed in the high-dose group (A3).
- In the ibuprofen-treated groups (Group B), platelet counts also demonstrated a decreasing trend, with the lowest mean value observed in the high-dose group (B4).
- Statistical analysis revealed a significant reduction in platelet counts in both high-dose groups compared with the control (*p* < 0.05, one-way ANOVA followed by LSD post-hoc test).

#### Lymphocytes (Lymph)

- In group C (control), lymphocyte counts were within the normal range.
- In the paracetamol-treated groups (Group A), lymphocyte counts showed a decreasing trend, with a significant reduction observed in the high-dose group (A3) (*p* < 0.05, one-way ANOVA followed by LSD post-hoc test).
- In the ibuprofen-treated groups (Group B), lymphocyte counts similarly decreased, with all groups showing a statistically significant reduction compared with the control (*p* < 0.05).

### Histopathological examination

Histopathological evaluation was performed as a qualitative descriptive assessment to identify early or gross tissue alterations following sub-chronic drug exposure. No semi-quantitative scoring system or lesion incidence analysis was applied, as the study was designed as an exploratory screening assessment rather than a definitive pathological grading study. Accordingly, all findings are presented as descriptive microscopic observations.

Histological evaluation was performed on coded slides to minimize observer bias.

Testes, liver, kidneys, and stomach tissues were collected from mice in all experimental groups (C, A, and B) and subjected to routine histopathological examination. The microscopic findings for each organ are presented in the following sections.

#### Histopathological examination of testes

Histopathological examination of testicular sections from the control group (C) revealed normal testicular architecture, with well-defined seminiferous tubules, an organized germinal epithelium, and complete spermatogenic series. Epididymal lumina were densely packed with spermatozoa.

**Fig. 1:**
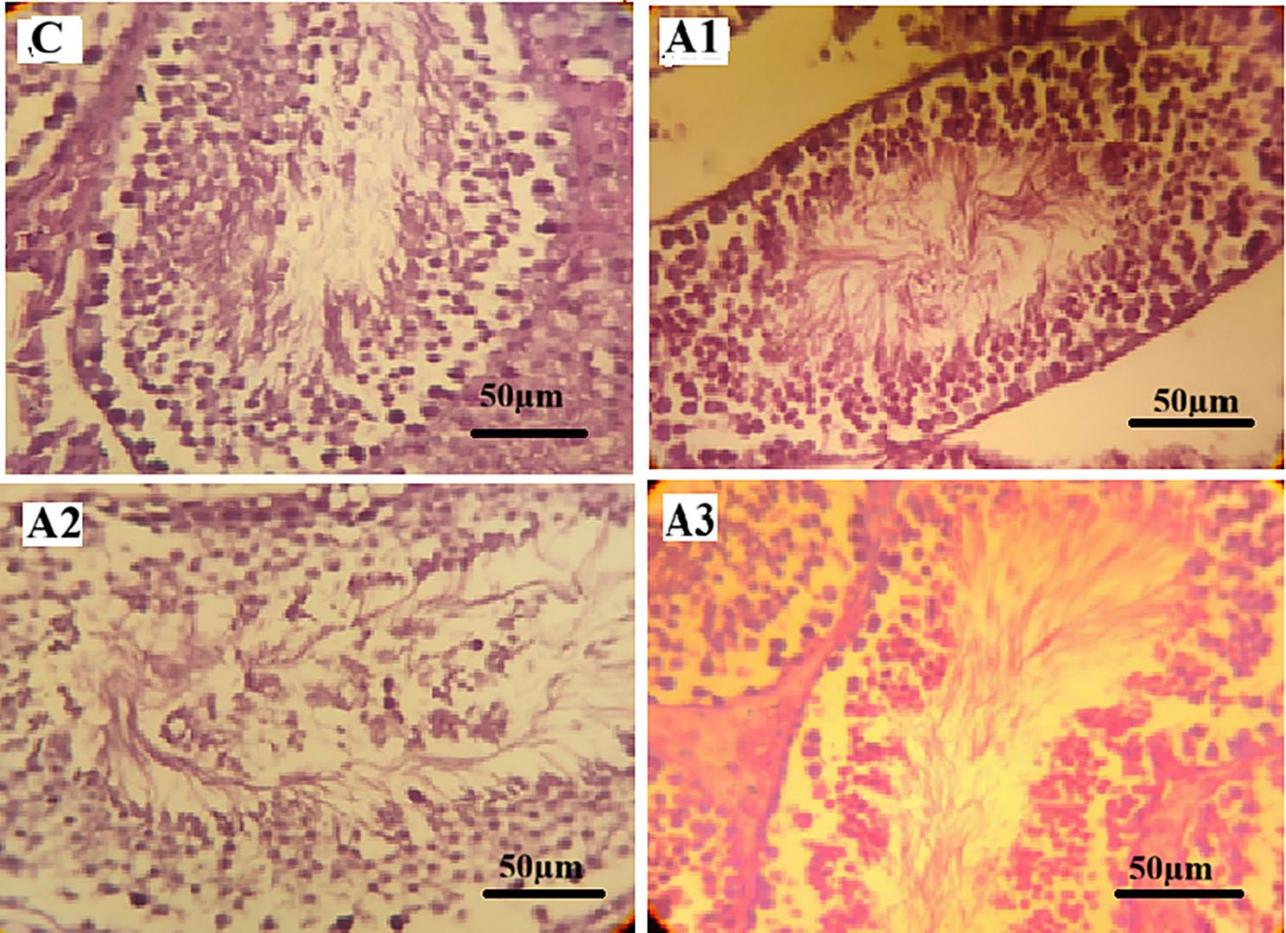
Photomicrographs of mice testicular tissue sections stained with Hematoxylin and Eosin (H&E, x400). (**C**) Control group showing normal seminiferous tubules with well-organized germinal epithelium and lumina containing abundant spermatozoa. (**A1**) Paracetamol-treated group showing largely preserved testicular architecture with organized germinal epithelium and ongoing spermatogenesis. (**A2**) Paracetamol-treated group showing partial disorganization of the germinal epithelium and loosening of spermatogenic cell layers. (**A3**) Paracetamol-treated group showing distortion of some seminiferous tubules, reduction of spermatogenic cell layers, and interstitial congestion. Scale bar = 50 μm

In paracetamol-treated groups (Fig. 1), histopathological alterations varied among the examined sections. Group A1 showed largely preserved seminiferous tubules with organized germinal epithelium and ongoing spermatogenesis, comparable to the control group. In Group A2, partial disorganization of the germinal epithelium and loosening of spermatogenic cell layers were observed. Group A3 exhibited structural alterations including distortion of some seminiferous tubules, reduction in spermatogenic cell layers, and interstitial congestion. Epididymal lumina contained spermatozoa in all paracetamol-treated groups.

**Fig. 2:**
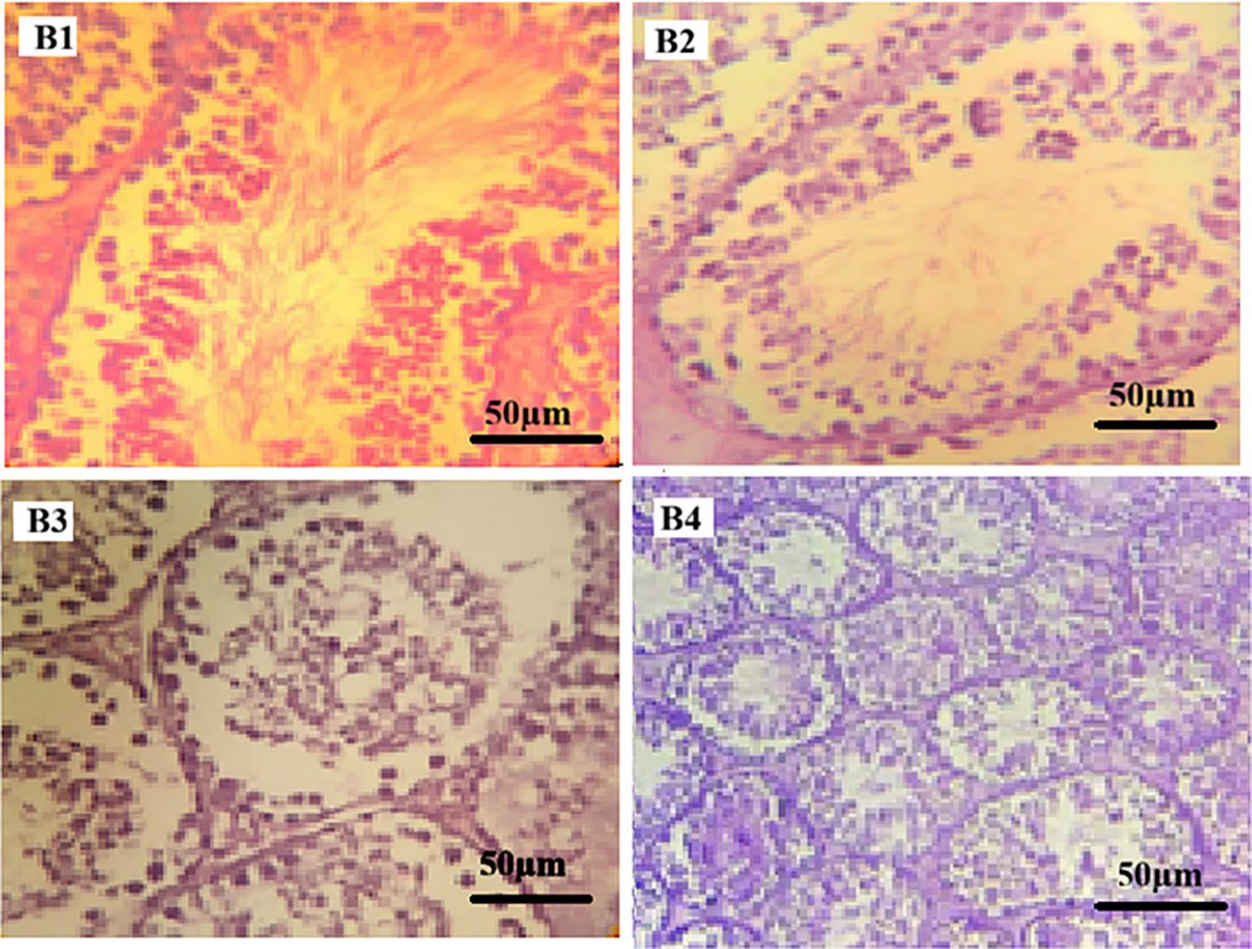
Photomicrographs of mice testicular tissue sections treated with Ibuprofen (H&E stain). Photomicrographs of mice testicular tissue sections treated with ibuprofen and stained with Hematoxylin and Eosin (H&E, ×400). (**B1**) Ibuprofen-treated group showing seminiferous tubules with mild disorganization of the germinal epithelium and focal interstitial congestion. (**B2**) Ibuprofen-treated group showing loosening of the germinal epithelium and widening of interstitial spaces. (**B3**) Ibuprofen-treated group showing sloughing of immature germinal cells into the tubular lumen and cytoplasmic vacuolization. (**B4**) Ibuprofen-treated group showing areas with reduced germinal cell layers and altered seminiferous tubular architecture. Scale bar = 50 μm

In ibuprofen-treated groups (Fig. 2), histopathological examination demonstrated structural alterations across the examined sections. Group B1 showed seminiferous tubules with mild disorganization of the germinal epithelium and focal interstitial congestion. In Group B2, loosening of germinal epithelium and widening of interstitial spaces were observed. Groups B3 and B4 exhibited alterations including sloughing of immature germinal cells into the tubular lumen, cytoplasmic vacuolization, and areas showing reduced germinal cell layers. Epididymal lumina contained spermatozoa in all ibuprofen-treated groups.

All testicular histopathological findings were evaluated qualitatively based on microscopic examination, without the application of a standardized semi-quantitative scoring system.

#### Histopathological examination of liver

Histopathological examination of liver sections from the control group (C) revealed normal hepatic architecture, characterized by well-arranged hepatocyte cords, intact portal areas, and preserved sinusoidal spaces.

**Fig. 3:**
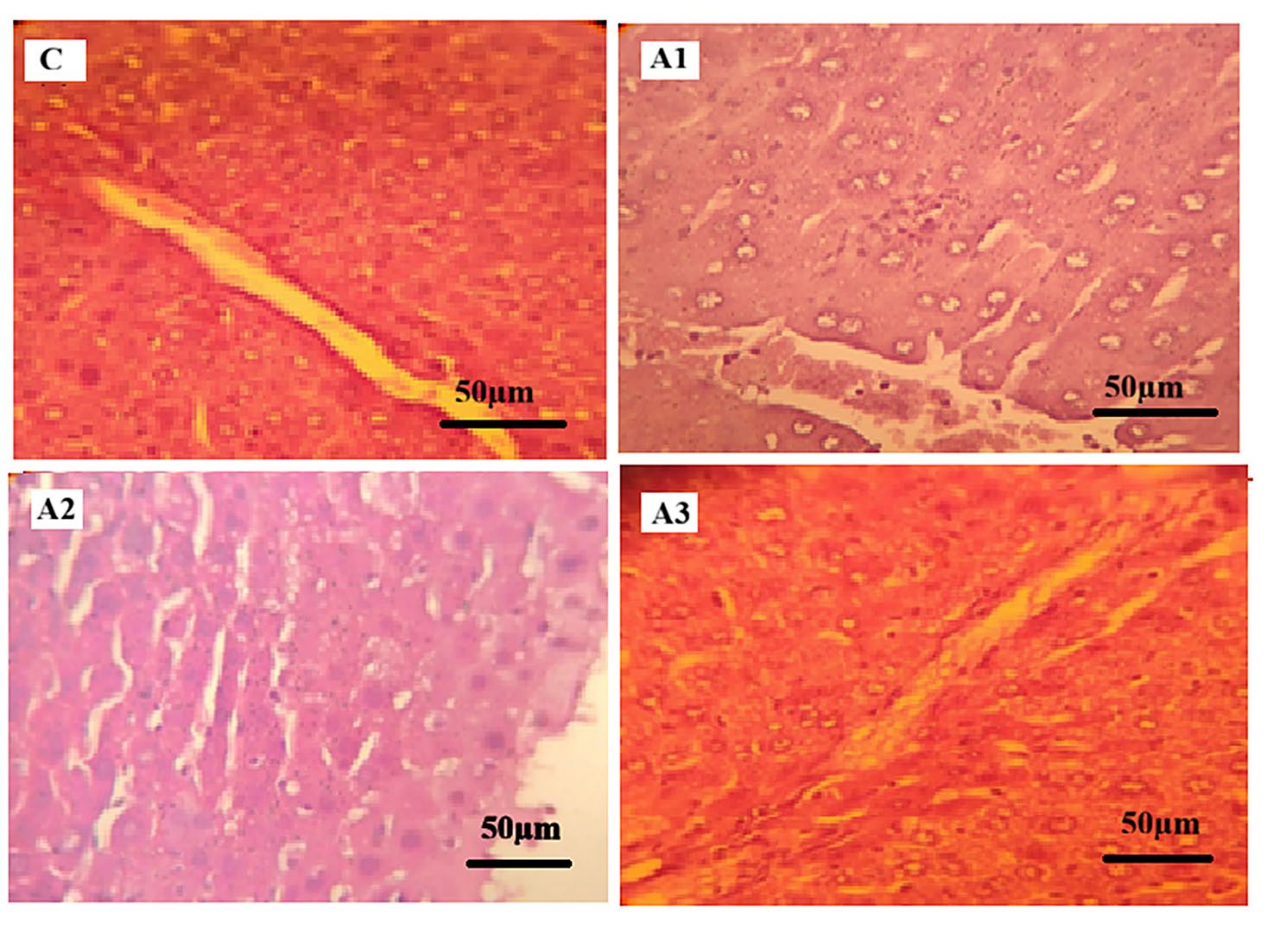
Photomicrographs of mice liver tissue sections stained with Hematoxylin and Eosin (H&E, x400). (**C**) Control group showing normal hepatic lobular architecture with a central vein and radiating hepatocyte cords. (**A1**) Paracetamol-treated group showing largely preserved hepatic architecture with granular cytoplasmic changes in hepatocytes. (**A2**) Paracetamol-treated group showing hepatocellular hydropic degeneration with cellular swelling and partial sinusoidal compression. (**A3**) Paracetamol-treated group showing focal areas of hepatocellular necrosis and vascular congestion. Scale bar = 50 μm

**Fig. 4:**
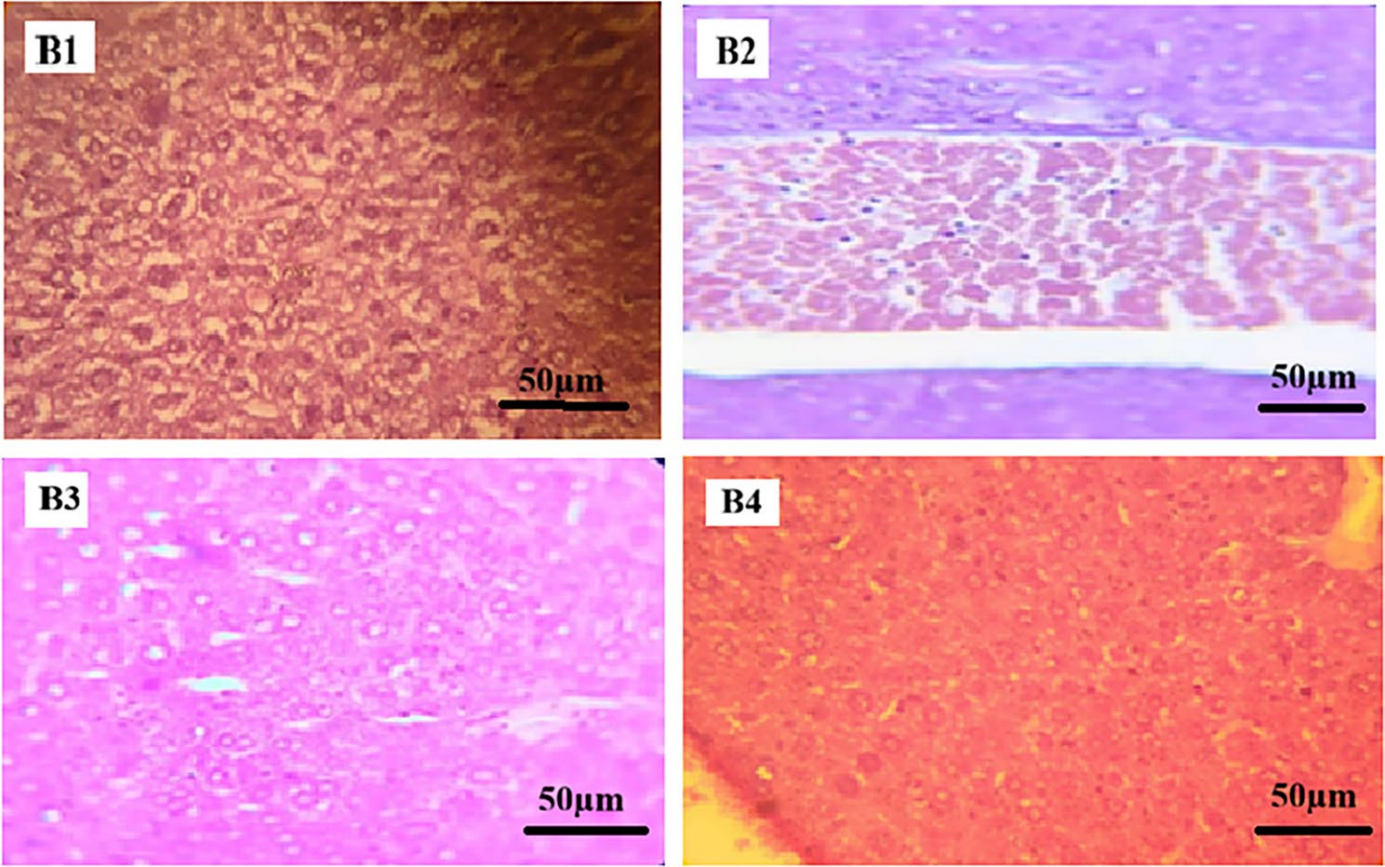
Photomicrographs of mice liver tissue sections treated Ibuprofen (H&E stain, x400). (**B1**) Ibuprofen-treated group showing preserved hepatic architecture with intact hepatocyte cords and sinusoidal spaces. (**B2**) Ibuprofen-treated group showing hepatic histology comparable to the control group. (**B3**) Ibuprofen-treated group showing hepatocellular hydropic degeneration with focal necrotic areas. (**B4**) Ibuprofen-treated group showing sinusoidal hemorrhage and disruption of the normal hepatic lobular architecture. Scale bar = 50 μm

In paracetamol-treated groups (Figs. 3 and 4), histopathological examination revealed structural alterations among the examined sections. Group A1 showed largely preserved hepatic architecture with mild granular cytoplasmic changes in hepatocytes. In Group A2, hepatocytes exhibited hydropic degeneration characterized by cellular swelling and cytoplasmic vacuolization, with partial sinusoidal compression. Group A3 demonstrated structural alterations including focal areas of hepatocellular necrosis and vascular congestion.

In ibuprofen-treated groups (Figs. 3 and 4), liver sections from Groups B1 and B2 displayed hepatic architecture comparable to the control group, with preserved hepatocyte arrangement and vascular structures. In Group B3, hepatocytes exhibited hydropic degeneration with focal necrotic areas. Group B4 showed histopathological alterations characterized by sinusoidal hemorrhage and disruption of the normal hepatic lobular organization.

All hepatic histopathological findings were assessed qualitatively based on microscopic examination, without the use of a standardized semi-quantitative scoring system.

#### Histopathological examination of the kidneys

Histopathological examination of kidney sections from the control group (C) revealed normal renal architecture, characterized by intact glomerular capillary loops, well-preserved tubular structures, and normal interstitial spaces.

**Fig. 5:**
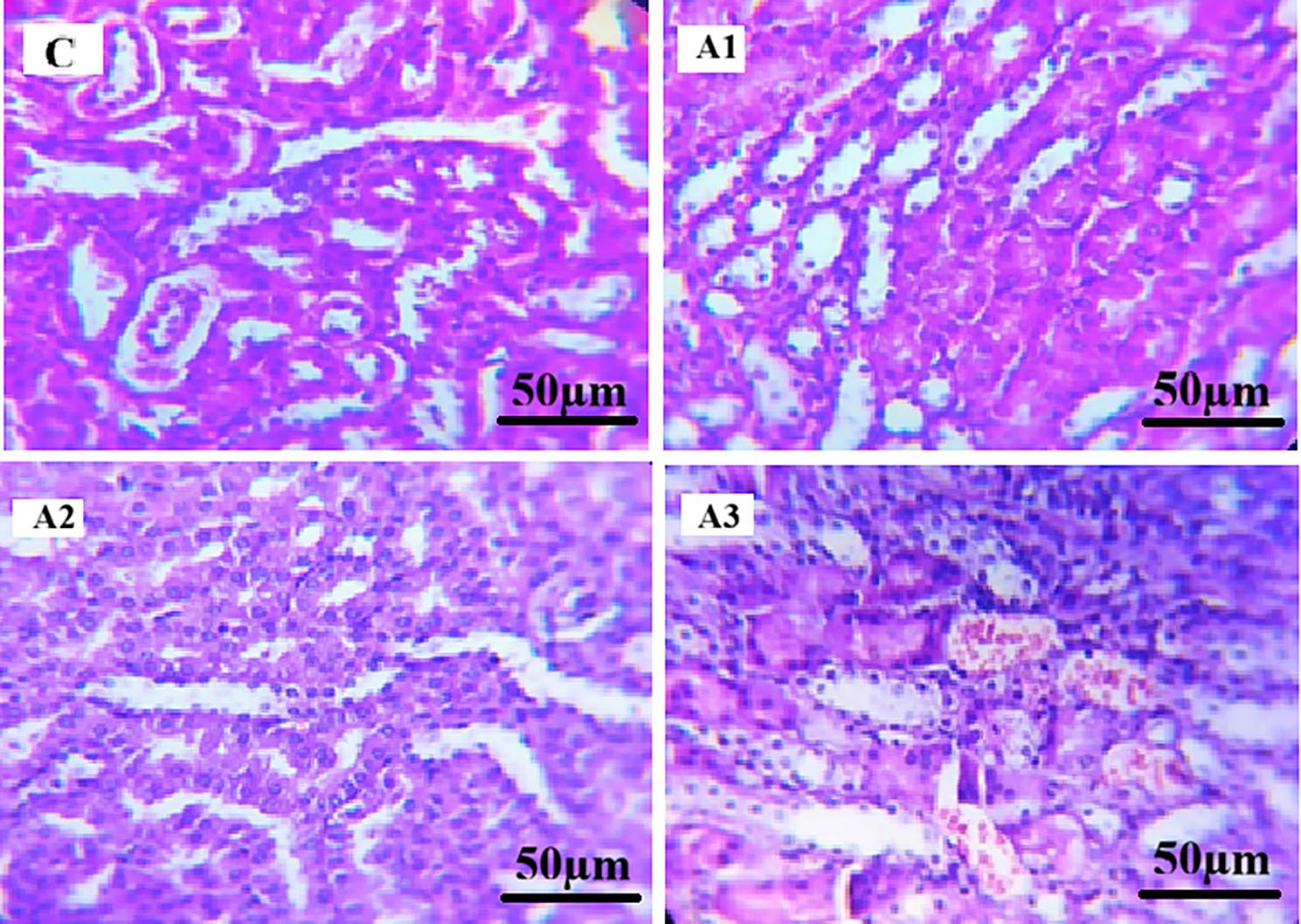
Photomicrographs of mice kidney tissue sections stained with Hematoxylin and Eosin (H&E, x400). (**C**) Control group showing normal renal architecture with intact glomeruli and renal tubules lined by well-preserved epithelium. (**A1**) Paracetamol-treated group showing largely preserved renal architecture with mild cloudy swelling of the tubular epithelium. (**A2**) Paracetamol-treated group showing tubular hydropic degeneration associated with narrowing of some tubular lumina. (**A3**) Paracetamol-treated group showing tubular necrotic changes with loss of tubular epithelial integrity and areas of interstitial congestion and hemorrhage. Scale bar = 50 μm

In paracetamol-treated groups (Fig. 5), histopathological examination demonstrated structural alterations among the examined sections. Group A1 showed largely preserved renal architecture with cloudy swelling of the tubular epithelial cells. In Group A2, tubular epithelial cells exhibited hydropic degeneration accompanied by narrowing of tubular lumina. Group A3 displayed structural alterations characterized by tubular necrotic changes, loss of tubular epithelial integrity, and increased interstitial congestion and focal hemorrhage.

**Fig. 6:**
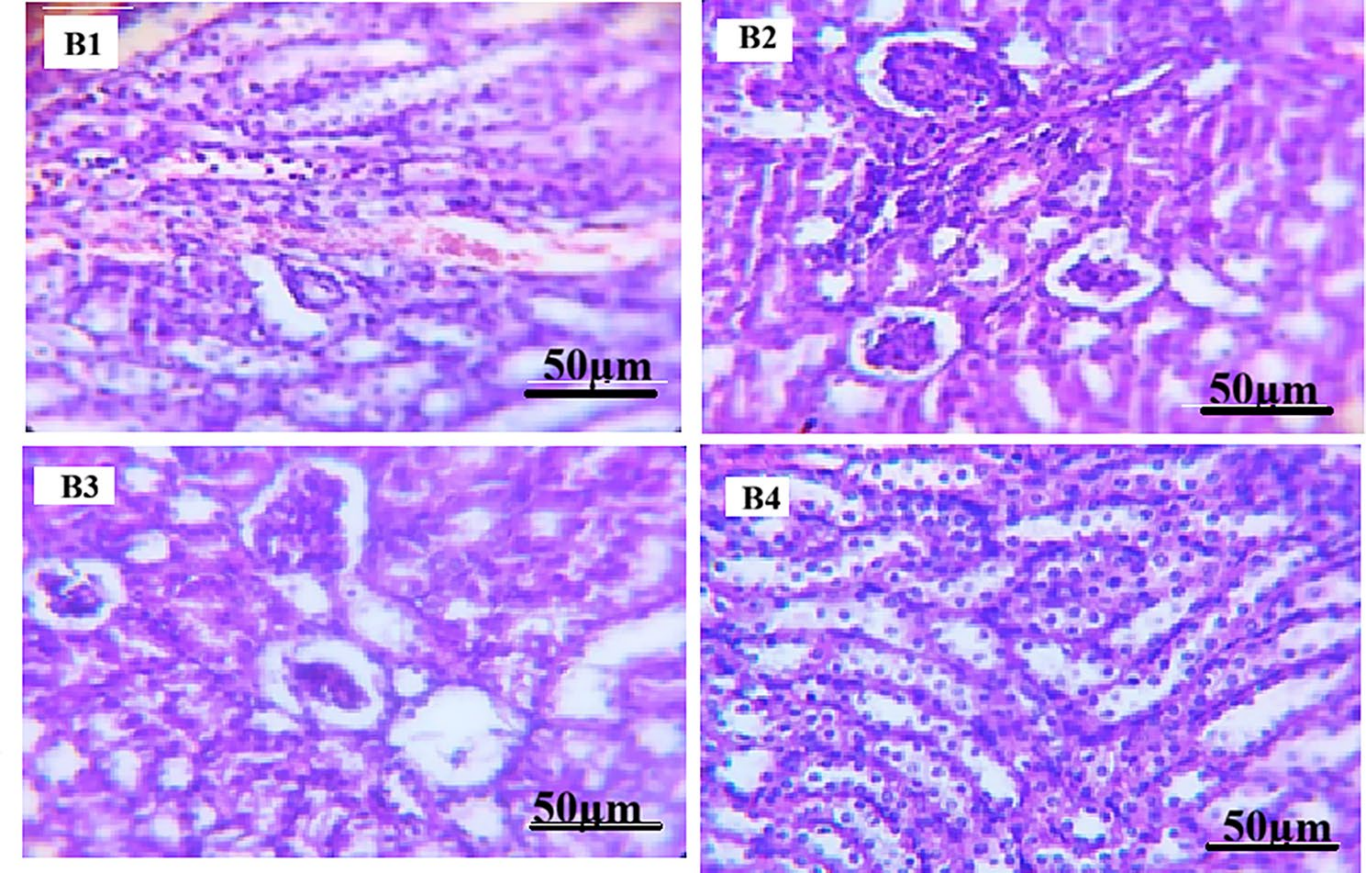
Photomicrographs of mice kidney tissue sections treated Ibuprofen (H&E stain, x400). (**B1**) Ibuprofen-treated group showing mild interstitial congestion with preserved glomerular and tubular architecture. (**B2**) Ibuprofen-treated group showing hydropic degeneration and swelling of renal tubular epithelium. (**B3**) Ibuprofen-treated group showing tubular necrotic changes with desquamation of epithelial cells and accumulation of cellular debris within tubular lumina. (**B4**) Ibuprofen-treated group showing tubular dilation with epithelial flattening/atrophy and the presence of intraluminal casts. Scale bar = 50 μm

In ibuprofen-treated groups (Fig. 6), renal sections from Group B1 showed preserved glomerular and tubular architecture with focal interstitial congestion. In Group B2, tubular epithelial cells exhibited hydropic degeneration and epithelial swelling. Group B3 demonstrated tubular injury characterized by necrosis with desquamation of epithelial cells and accumulation of cellular debris within tubular lumina. Group B4 exhibited histopathological alterations including tubular dilation, epithelial flattening, and the presence of intraluminal casts.

All renal histopathological findings were evaluated qualitatively based on microscopic examination, and representative photomicrographs are presented in Figs. 5 and 6.

#### Histopathological examination of the stomach

Histopathological examination of stomach sections from the control group (C) revealed intact gastric mucosa with preserved glandular architecture and a continuous epithelial lining.

**Fig. 7:**
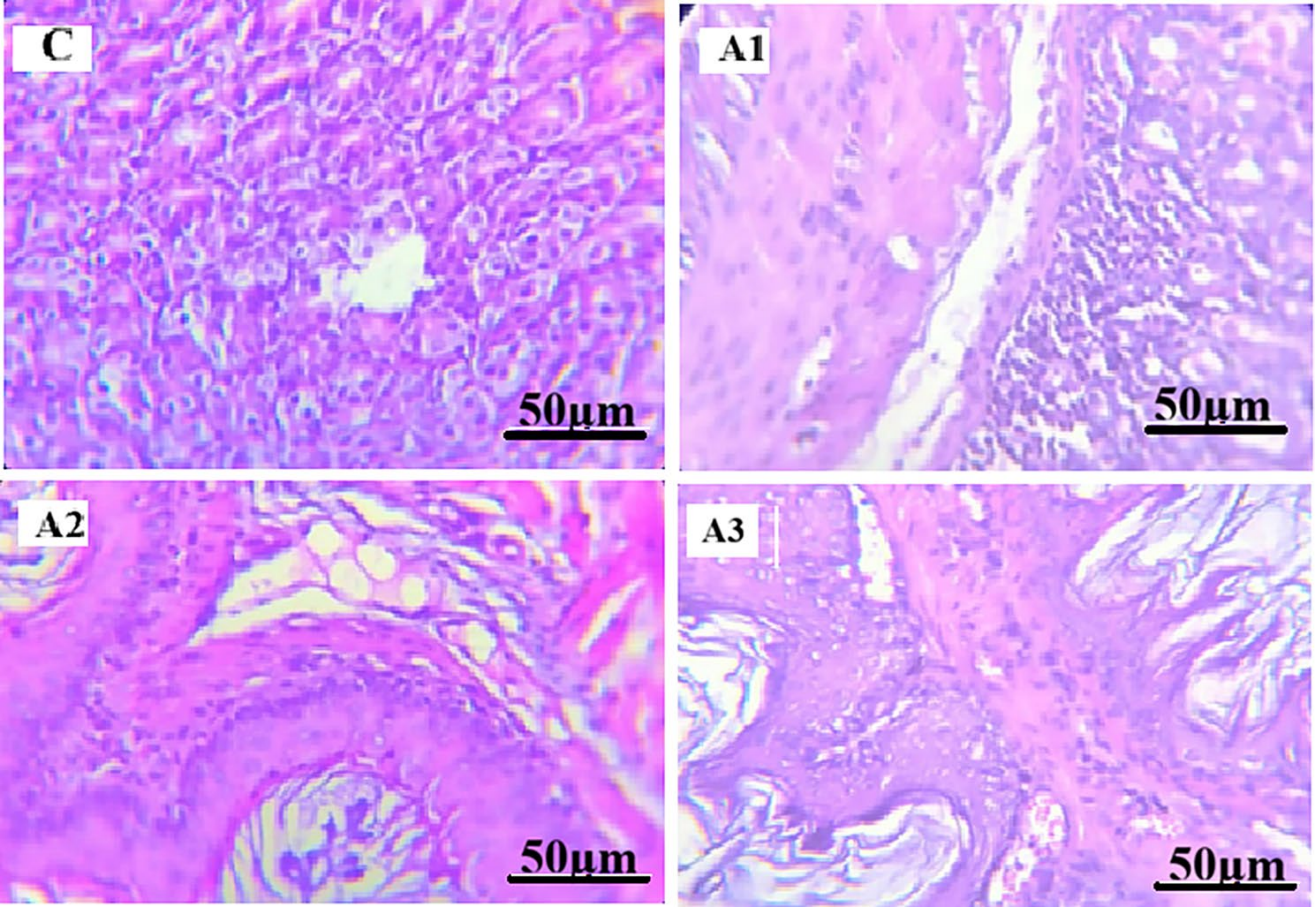
Photomicrographs of mice stomach tissue sections stained with Hematoxylin and Eosin (H&E, x400). (**C**) Control group showing intact gastric mucosa with preserved glandular architecture and continuous epithelial lining. (**A1**) Paracetamol-treated group showing mild superficial mucosal erosion with limited epithelial disruption. (**A2**) Paracetamol-treated group showing submucosal edema (clear spaces), glandular distortion, and epithelial shedding. (**A3**) Paracetamol-treated group showing mucosal ulceration, epithelial necrosis, and loss of normal glandular architecture. Scale bar = 50 μm

In paracetamol-treated groups (Fig. 7), histopathological examination demonstrated structural alterations among the examined sections. Group A1 showed superficial mucosal erosion with limited epithelial disruption. In Group A2, gastric sections exhibited submucosal edema and glandular distortion accompanied by epithelial shedding. Group A3 displayed histopathological alterations characterized by mucosal ulceration, epithelial necrosis, and loss of normal glandular architecture.

**Fig. 8:**
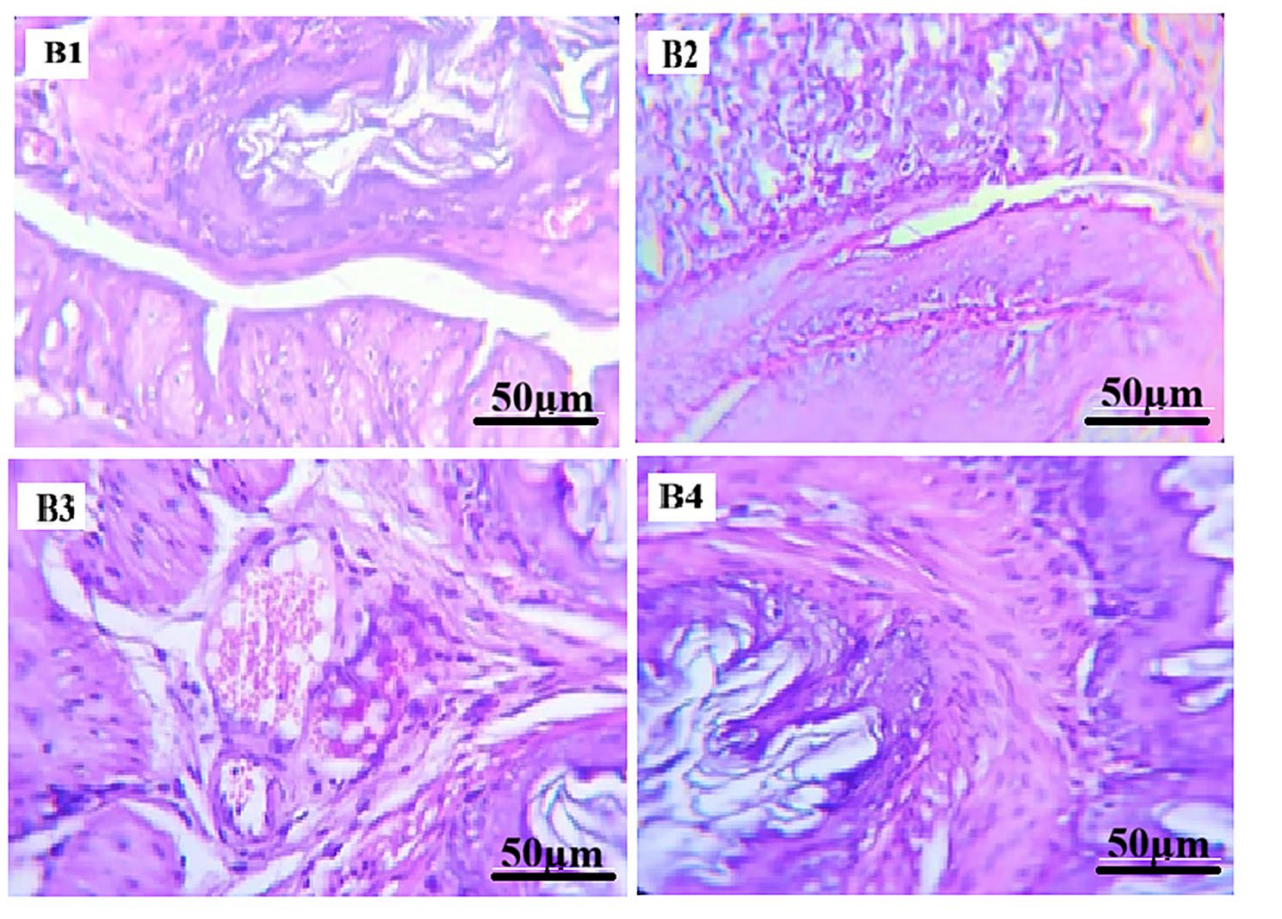
Photomicrographs of mice stomach tissue sections treated with Ibuprofen (H&E stain, x400). (**B1**) Ibuprofen-treated group showing mild mucosal surface alterations with preserved glandular structure. (**B2**) Ibuprofen-treated group showing epithelial desquamation accompanied by submucosal edema. (**B3**) Ibuprofen-treated group showing vascular congestion and hemorrhage associated with mucosal erosion. (**B4**) Ibuprofen-treated group showing extensive mucosal ulceration with epithelial necrosis and disruption of normal mucosal architecture. Scale bar = 50 μm

In ibuprofen-treated groups (Fig. 8), stomach sections from Group B1 showed mild surface epithelial alterations with largely preserved glandular structure. In Group B2, epithelial desquamation with submucosal edema was observed. Group B3 exhibited vascular congestion and hemorrhage associated with mucosal erosion. Group B4 demonstrated extensive mucosal ulceration with epithelial necrosis and disruption of normal mucosal architecture.

All gastric histopathological findings were assessed qualitatively based on microscopic examination, without the application of a standardized numerical grading or semi-quantitative scoring system. Representative photomicrographs are shown in Figs. 7 and 8.

### Molecular analysis

#### TNF-ɑ & connexin genes expression analysis

Total RNA was extracted from blood samples of groups A, B, and C. Gene expression of TNF-α and connexin 43 was then analyzed by qRT-PCR to assess the molecular effects induced by paracetamol and ibuprofen treatments. The obtained results of these were as follows:

##### Real-time PCR result

**Fig. 9:**
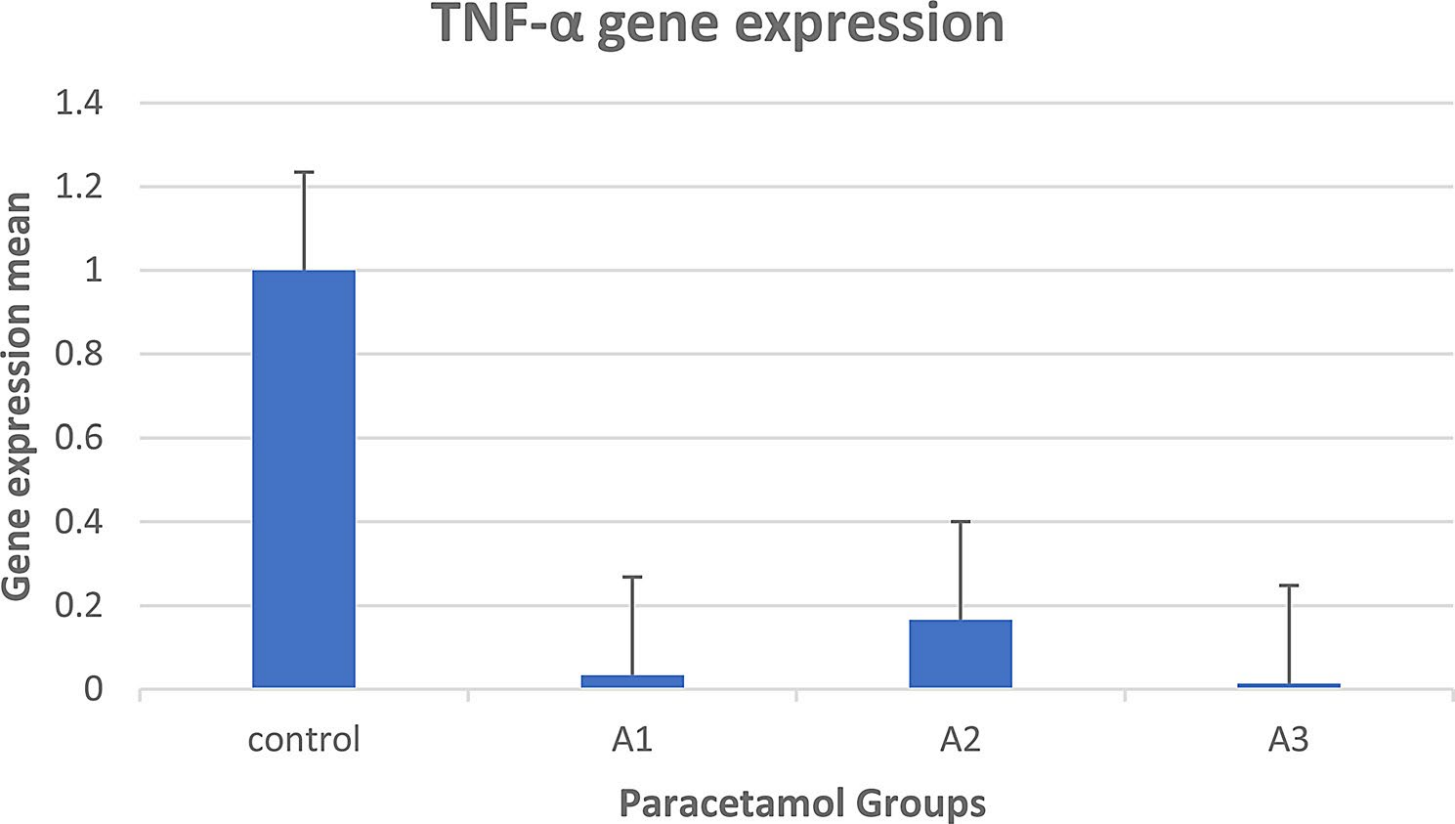
Mean gene expression of the TNF gene after paracetamol treatment for groups C (control) and A (**A1**, **A2**, and **A3**)

In group A (paracetamol treatment), it was shown that the mean gene expression of the TNF gene was downregulated after paracetamol treatment in groups C (control) and A (A1, A2, and A3), as shown in Fig. 9.

**Fig. 10:**
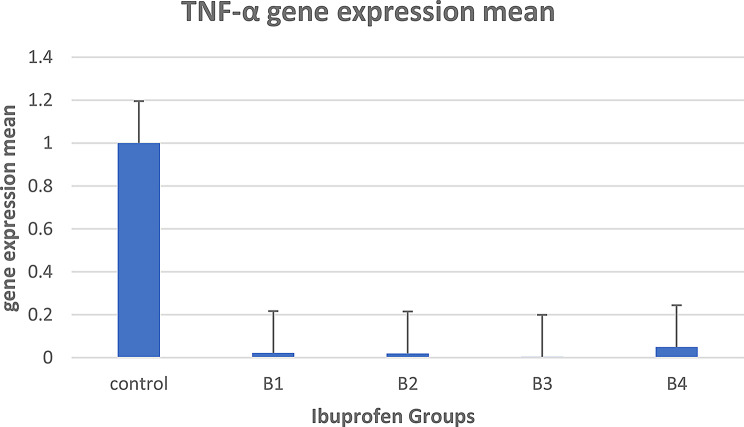
Mean gene expression of the TNF gene after ibuprofen treatment for groups C (control) and B (**B1**, **B2**, **B3**, and **B4**)

In group B (ibuprofen treatment), it was shown that the mean gene expression of the TNF gene was downregulated after ibuprofen treatment in groups C (control) and B (B1, B2, B3, and B4). Figure 10 illustrates these results.

**Fig. 11:**
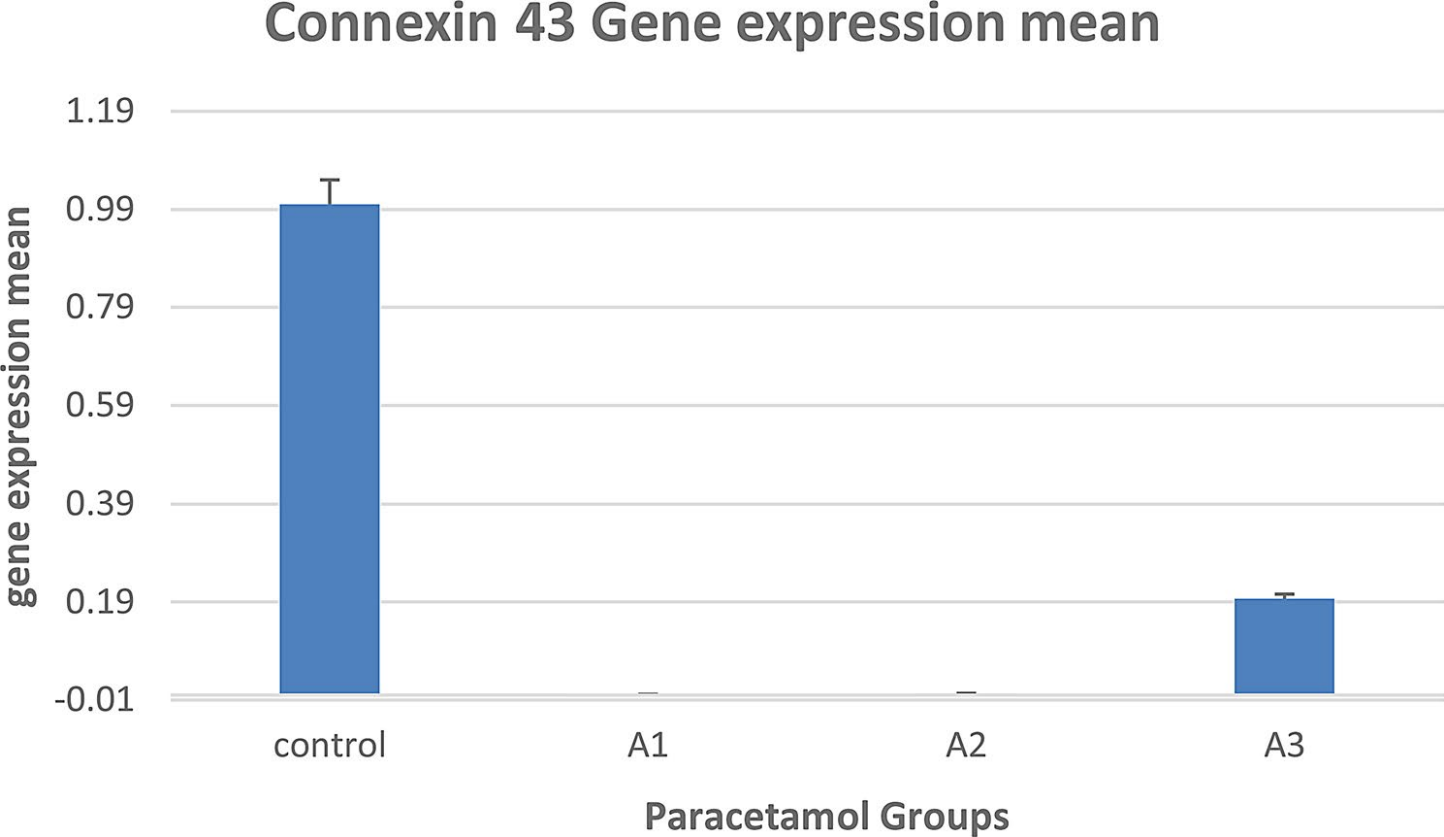
Mean gene expression of gene connexin 43 after paracetamol treatment for groups C (control) and A (**A1**, **A2**, and **A3**)

In group A (paracetamol treatment), it was shown that the mean gene expression of gene connexin 43 was downregulated after paracetamol treatment in groups C (control) and A (A1, A2, and A3), as presented in Fig. 11.

**Fig. 12:**
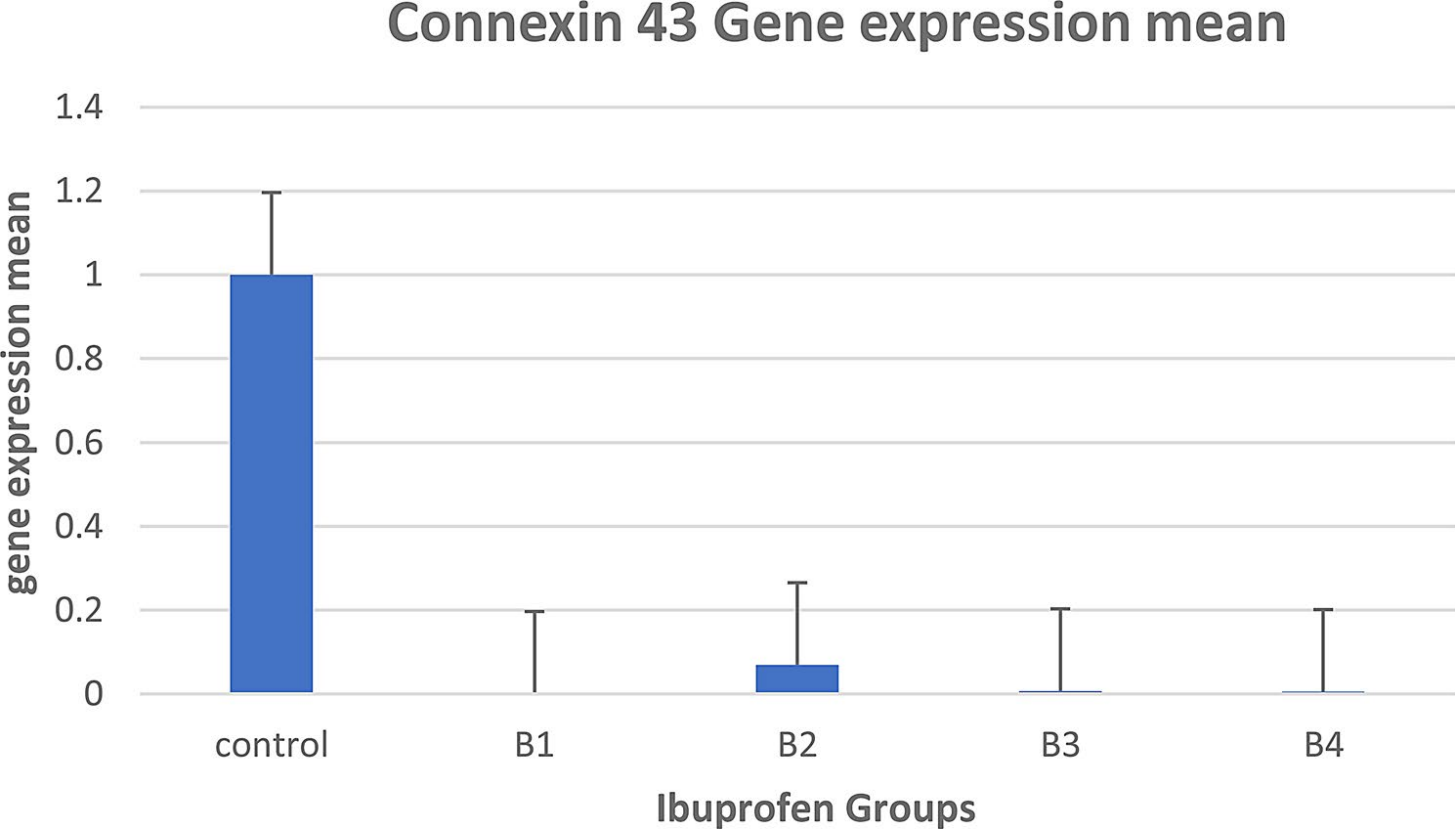
Mean gene expression of gene connexin 43 after ibuprofen treatment for groups C (control) and B (**B1**, **B2**, **B3**, and **B4**)

In group B (ibuprofen treatment), it was shown that the mean gene expression of gene connexin 43 was downregulated after ibuprofen treatment, in group C (control) and group B (B1, B2, B3, and B4), Fig. 12 shows these findings.

## Discussion

In the present study, male albino mice were used to investigate the hematological, histopathological, and molecular responses to subchronic exposure to the antipyretic drugs paracetamol and ibuprofen. Hematological evaluation revealed alterations in selected complete blood count (CBC) parameters, while histological examination demonstrated dose-related structural changes in the liver, kidneys, stomach, and testes.

In addition, qRT-PCR analysis of blood-derived RNA demonstrated a reduction in the mRNA expression levels of connexin 43 (Cx43) and tumor necrosis factor-alpha (TNF-α) in both paracetamol-treated (groups A1–A3) and ibuprofen-treated (groups B1–B4) mice compared with the control group. These changes reflect alterations in circulating gene expression profiles and may represent systemic molecular responses to drug exposure rather than direct tissue-specific effects.

The observed alterations in hematological parameters correlate with mild histopathological changes in liver and kidney tissues, suggesting early subclinical effects of paracetamol overdose. This connection supports the notion that hematology can serve as a sensitive early indicator before overt tissue pathology manifests.

However, the biological implications of TNF-α and connexin 43 downregulation observed at the mRNA level should be interpreted cautiously, as transcriptional changes do not necessarily correspond to alterations in protein expression or cellular function.

Experimental animals were divided randomly into three groups: Group A, paracetamol (A1: 12.5 mg/kg/day), A2 (25 mg/kg/day), and A3 (50 mg/kg/day). Group B, ibuprofen (B1 10 mg/kg/day, B2 20 mg/kg/day, B3 30 mg/kg/day, and B4 50 mg/kg/day) and Group C (control).

Firstly, the animal groups were given antipyretics (paracetamol and ibuprofen) in the doses mentioned in all separate animal groups (five mice for every group) orally every day for a whole month. Secondly, animal groups A, B, and C were examined to find out the assessment of micro- and macro-DNA lesions induced by some antipyretics for 30 days.

Although there were variations between the three groups (A, B, and C) before and after receiving ibuprofen and paracetamol treatments, the statistical computations revealed that the drop in body weights was not significant. This observation may reflect nonspecific physiological responses to repeated drug exposure, although the exact mechanism cannot be determined from the current data, when taking both medications. This is consistent with a previous study by [35], whereby albino male mice were administered different dosages of paracetamol for 42 days. The findings indicated that the mouse’s body weight appeared to decrease in comparison to the control group. When albino male mice were given different doses of paracetamol for 42 days, their body weight changed in comparison to the control group, according to the study’s body mass indices.

The experiment demonstrated a mild effect on blood components, including red blood cells, hemoglobin, platelets, and white blood cells, these alterations may indicate early changes in hematological profiles, but their clinical significance cannot be determined in this study. After completing the statistical calculations, the changes in RBCs and HBG were not significant, where differences were found among the three groups (A, B, and C) after treatment. Analysis also showed significant changes in WBCs and lymphocytes only in ibuprofen treatments. However, a significant lymph decrease in the highest paracetamol dose (A3) was observed. However, there was a substantial difference in PLT levels between the two groups. These findings suggest differential hematological responses between ibuprofen- and paracetamol-treated groups, which should be interpreted cautiously.

In a study by [36] Paracetamol had less adverse effects than both indomethacin and ibuprofen, primarily on renal function, platelet count, and GIT hemorrhage, but is just as effective on PLT in closing hs-PDA in premature neonates. This is consistent with the outcomes of paracetamol.

In mice, ibuprofen and paracetamol have hematological and immunomodulatory effects. which were examined by [37]. The findings demonstrated that, in comparison to the control, taking these medications every day for a month may be associated with alterations in selected hematological parameters. Both ibuprofen and paracetamol have been reported to induce structural and histological alterations in different organs when used excessively or for extended periods. In the present study, mild histological alterations were observed in some treated groups.

The present study evaluated the histopathological effects of paracetamol and ibuprofen on the testes, liver, kidneys, and stomach of male mice following repeated exposure. The findings demonstrated organ-specific structural alterations of varying severity, indicating that both drugs can induce tissue changes even in the absence of quantitative histological scoring.

Histopathological examination of the testes revealed progressive structural alterations in treated groups, including disorganization of the germinal epithelium, loosening and sloughing of spermatogenic cells, cytoplasmic vacuolization, and focal tubular atrophy, while epididymal lumina generally retained spermatozoa. These observations align with previous reports indicating that commonly used analgesics such as paracetamol and ibuprofen can adversely affect male reproductive tissues, potentially impairing spermatogenesis without producing overt clinical lesions [38]. Additionally, experimental studies have demonstrated that non-steroidal anti-inflammatory drugs may alter reproductive function and sperm parameters even when gross histological damage is limited [39]. Such findings suggest that structural preservation does not necessarily exclude functional impairment of the male reproductive system.

Liver sections from treated groups showed a spectrum of histopathological alterations ranging from mild hepatocellular granular changes and hydropic degeneration to focal necrosis, vascular congestion, and sinusoidal hemorrhage in more affected groups. These findings are consistent with the well-documented hepatotoxic potential of paracetamol and, to a lesser extent, ibuprofen. Paracetamol-induced liver injury remains a leading cause of acute liver failure worldwide, particularly following overdose or prolonged exposure [40]. Experimental toxicological studies have also reported hepatic alterations following ibuprofen administration, including inflammatory changes and vascular disturbances, which may be influenced by both dose and duration of exposure [41]. In the present study, the observed hepatic lesions were assessed qualitatively, and therefore, comparisons of severity should be interpreted cautiously.

Kidney tissues exhibited histopathological changes including tubular cloudy swelling, hydropic degeneration, epithelial desquamation, tubular necrosis, interstitial congestion, hemorrhage, and intraluminal cast formation in more affected groups. These findings are in agreement with previous studies reporting that non-steroidal anti-inflammatory drugs can induce renal tubular injury and interstitial alterations, particularly with repeated exposure [42]. Although hepatotoxicity is more commonly associated with paracetamol, renal damage and acute renal failure have been reported independently of hepatic injury [43]. The present renal findings highlight the susceptibility of renal tubular structures to analgesic-induced toxicity.

Histopathological examination of stomach tissues revealed mucosal erosion, epithelial shedding, submucosal edema, vascular congestion, hemorrhage, and ulceration in treated groups, with increasing severity in more affected specimens. These alterations are consistent with the well-established gastrointestinal toxicity of ibuprofen and other NSAIDs, which can compromise gastric mucosal integrity and lead to erosive or ulcerative lesions. Paracetamol-related gastric changes were generally milder, in agreement with its comparatively lower direct gastric irritant effect; however, structural mucosal alterations have been reported under certain exposure conditions [44].

These observations are consistent with previous experimental studies indicating that reproductive tissues may exhibit early structural alterations following pharmacological exposure, even before overt functional impairment becomes evident. Moreover, NSAID-induced reproductive toxicity, including gonadal tissue injury mediated through inflammatory and molecular pathways, has been previously reported, supporting the relevance of the present findings [45].

According to a review, using NSAIDs is associated with a number of GI issues, including stomach ulcers, bleeding, and perforation [46]. The study recommends performing comprehensive risk-benefit analyses before taking NSAIDs and emphasizes that high-risk patients are more likely to have these side effects.

Additionally, a study looked into NSAID-induced gastrointestinal hemorrhage in older people [47]. The findings indicate that long-term NSAID use combined with risk factors such as a history of ulcers or concomitant use of contraceptives significantly increases the chance of serious gastrointestinal bleeding.

Fas-induced liver damage results from the production of tumor necrosis factor (TNF) by macrophages following the stimulation of neutrophils by Fas activation.

Gene expression analysis was conducted using peripheral blood samples and therefore reflects systemic molecular responses to repeated drug exposure rather than tissue-specific regulatory mechanisms within individual organs. Accordingly, the observed changes in TNF-α and Connexin 43 expression should be interpreted as systemic transcriptional responses rather than direct indicators of molecular alterations within specific tissues.

Paracetamol was associated with a systemic reduction in pro-inflammatory cytokine levels, such as TNF-alpha, following many treatments. This decrease in TNF-alpha is consistent with its role in controlling inflammatory processes and raises the possibility that it could help lessen excessive inflammation.

This result is in agreement with a previous study by [48]; Mice that had been fasting for 24 h were challenged with the agonistic Jo2 anti-Fas antibody. They were then given either saline or ibuprofen, a dual COX-1 and COX-2 inhibitor. Both the Jo2-mediated stimulation of myeloperoxidase-secreting neutrophils and macrophages in the liver and the rise in serum TNF were decreased by ibuprofen. Ibuprofen also decreased hepatic glutathione depletion, bid truncation, caspase activation, outer mitochondrial membrane rupture, and hepatocyte apoptosis in order to lower mice’s mortality later on [49], Previous studies have reported that high concentrations of ibuprofen are associated with reduced cellular viability and proliferation in experimental models, roughly 500 µM. Additionally, it was found that ibuprofen efficiently decreased TNF-alpha at modest dosages. Marked downregulation of TNF-α may reflect an early adaptive or regulatory transcriptional response to repeated drug exposure. Given the multifaceted role of TNF-α in immune homeostasis, such changes should be interpreted cautiously without inferring functional immune impairment. It is important to emphasize that TNF-α was assessed at the mRNA level only, and no conclusions regarding cytokine protein levels or functional immune outcomes can be drawn from the present data.

About the Connexin43 gene expression [50], **C**onnexin 43 (Cx43) is a widely expressed gap junction protein that plays an essential role in intercellular communication and the maintenance of tissue homeostasis. Alterations in Cx43 expression have been reported in various pathological conditions, including inflammatory and toxicological contexts, where disrupted gap junction communication may contribute to altered cellular responses.

Experimental and clinical studies have suggested that dysregulation of Cx43 expression is associated with processes such as inflammation, oxidative stress, and cell death signaling. However, the functional consequences of these changes are highly context-dependent and may vary according to tissue type, exposure duration, and regulatory mechanisms.

In the present study, changes in Cx43 mRNA expression were assessed in peripheral blood as an indicator of systemic molecular responses to repeated drug exposure. These findings should be interpreted cautiously, as transcriptional alterations in circulating cells do not necessarily reflect tissue-specific regulation or functional outcomes in target organs. Further studies involving tissue-level and protein-based analyses would be required to clarify the precise role of Cx43 in drug-induced toxicity [50]. Accordingly, the observed changes in Cx43 mRNA expression should be interpreted as transcriptional modulation rather than evidence of altered gap junction protein function.

Ibuprofen administration attenuates serum TNF-α levels, hepatic glutathione depletion, hepatic apoptosis and mouse mortality after Fas stimulation [51].

Accordingly, the administered doses should be interpreted within the context of an experimental sub-chronic exposure model rather than as direct equivalents of human therapeutic use. Where the administered doses were selected based on previously published rodent toxicology studies and were designed to model sub-chronic exposure rather than acute overdose or therapeutic dosing in humans. The observed findings therefore reflect early toxicological and adaptive responses under repeated exposure conditions.

## Limitations

This study has several limitations. The sample size was relatively small (*n* = 5 per subgroup), which may limit the statistical power and the generalizability of the findings. Gene expression analyses were performed on peripheral blood rather than on specific target tissues such as the liver, kidney, stomach, or testes, and therefore may not fully reflect tissue-level molecular responses. The exposure duration was limited to 30 days, which does not allow assessment of chronic or long-term toxic effects. The use of commercial tablet formulations rather than pure active compounds may introduce variability due to pharmaceutical excipients. Functional biochemical markers such as liver and kidney function tests were not evaluated, which restricts interpretation of organ performance beyond histological observations. Only male mice were included, preventing sex-related comparisons. Histopathological evaluation was qualitative, without the application of a blinded semi-quantitative scoring system, and serum biochemical markers were not assessed. Consequently, the observed histological alterations should be interpreted as structural changes rather than definitive evidence of functional impairment. Peripheral blood mRNA levels are surrogate markers and may not directly correspond to organ-specific expression of TNF-α or Cx43. Statistical analysis was not blinded, which is acknowledged as a limitation. The human-equivalent doses used in this study are provided for comparative context only and do not reflect high-dose or overdose scenarios, nor do they directly replicate real-world human therapeutic dosing patterns. Importantly, the present findings should be interpreted within the limitations of the study, and the observed molecular and histological changes likely represent early or adaptive systemic responses to repeated drug exposure rather than definitive pathological outcomes.

## Conclusion

This study demonstrates that repeated sub-chronic exposure to paracetamol and ibuprofen at human-relevant exposure ranges (HED-based), not intended to model clinical therapeutic dosing doses induces measurable hematological, histological, and molecular alterations in male albino mice. Hematological changes were more pronounced in ibuprofen-treated groups, whereas paracetamol produced relatively milder effects. The observed reductions in selected blood parameters may reflect early modulation of immune-related indices rather than overt immune dysfunction.

Qualitative histopathological examination revealed structural alterations in the testes, liver, kidneys, and stomach compared with controls, including disrupted spermatogenesis and degenerative or inflammatory changes in other tissues, indicating differential tissue susceptibility. These alterations may represent early structural changes preceding functional impairment, particularly in the absence of quantitative histopathological assessment.

At the molecular level, repeated exposure was associated with downregulation of TNF-α and connexin 43 mRNA expression in peripheral blood, suggesting early systemic transcriptional responses without direct evidence of tissue-specific or protein-level functional effects. Overall, these findings should be regarded as preliminary and highlight the need for further studies incorporating quantitative histopathological analysis, tissue-specific molecular investigations, and functional biochemical assessments.

Despite the acknowledged methodological limitations including the use of peripheral blood for gene expression analysis and the absence of direct mechanistic validation, the present study provides a valuable integrated toxicological assessment of two widely used analgesics under sub-chronic exposure conditions. By combining hematological profiling, multi-organ histopathology, and systemic transcriptional analysis within a single experimental framework, this work contributes comparative insight into early biological responses to repeated therapeutic dosing of paracetamol and ibuprofen. The identification of coordinated hematological alterations alongside modulation of TNF-α and Connexin 43 expression suggests that measurable systemic responses may precede overt organ damage. Collectively, these findings extend current understanding of early adaptive molecular and hematological perturbations associated with prolonged analgesic exposure and provide a structured foundation for future mechanistic and tissue-specific investigations.

## Data Availability

All data generated or analyzed during this study are included in this published article and its supplementary information files. Additional datasets are available from the corresponding author upon reasonable request.

## Abbreviations

TNF-α: Tumor Necrosis Factor alpha
Cx43: Connexin 43
DNA: Deoxyribonucleic Acid
EDTA: Ethylenediaminetetraacetic acid
kg: Kilogram
g: Gram
mg: Milligram
µg: Microgram
ml: Milliliter
µl: Microliter
CBC: Complete Blood Count
RBC: Red Blood Cells
WBC: White Blood Cells
HGB: Hemoglobin
PLT: Platelet
Lymph: Lymphocytes
w/w: Weight per Weight
mRNA: Messenger RNA
cDNA: Complementary DNA
qRT-PCR: Quantitative Real-Time PCR
TNF-α: Tumor Necrosis Factor-alpha
GAPDH: Glyceraldehyde-3-phosphate dehydrogenase

## References

[1] Przybyła GW, Szychowski KA, Gmiński J. Paracetamol – An old drug with new mechanisms of action. Clin Exp Pharmacol Physiol. 2021;48(1):3–19. 32767405 10.1111/1440-1681.13392

[2] Anderson BJ. Paracetamol (Acetaminophen): mechanisms of action. Paediatr Anaesth. 2008;18(10):915–21. 18811827 10.1111/j.1460-9592.2008.02764.x

[3] McGill MR, Sharpe MR, Williams CD, Taha M, Curry SC, Jaeschke H. The mechanism underlying acetaminophen-induced hepatotoxicity in humans and mice involves mitochondrial damage and nuclear DNA fragmentation. J Clin Invest. 2012;122(4):1574–83. 22378043 10.1172/JCI59755 PMC3314460

[4] Alchin J, Dhar A, Siddiqui K, Christo PJ. Why paracetamol (acetaminophen) is a suitable first choice for treating mild to moderate acute pain in adults with liver, kidney or cardiovascular disease, gastrointestinal disorders, asthma, or who are older. Curr Med Res Opin. 2022;38(5):811–25. 35253560 10.1080/03007995.2022.2049551

[5] Ahmed HM, Shehata HH, Mohamed GS, Abo-Gabal HH, El-Daly SM. Paracetamol overdose induces acute liver injury accompanied by oxidative stress and inflammation. Egypt J Chem. 2023;66(3):399–408.

[6] Bauer AZ, Swan SH, Kriebel D, Liew Z, Taylor HS, Bornehag CG, et al. Paracetamol use during pregnancy – a call for precautionary action. Nat Rev Endocrinol. 2021;17(12):757–66. 34556849 10.1038/s41574-021-00553-7 PMC8580820

[7] Mohamed NA, Hassan MH, Saleem TH, Mohamed SA, El-Zeftawy M, Ahmed EA, et al. KIM-1 and GADD153 gene expression in paracetamol-induced acute kidney injury: effects of N-acetylcysteine, N-acetylmethionine, and N-acetylglucosamine. Turk J Biochem. 2022;47(4):409–16.

[8] Ding R, Liu S, He C, Nie X. Paracetamol affects the expression of detoxification- and reproduction-related genes and alters the life traits of Daphnia magna. Ecotoxicology. 2020;29(4):398–406. 32300985 10.1007/s10646-020-02199-z

[9] Koehn LM, Huang Y, Habgood MD, Kysenius K, Crouch PJ, Dziegielewska KM, Saunders NR. Effects of paracetamol (acetaminophen) on gene expression and permeability properties of the rat placenta and fetal brain. F1000Research. 2020;9:573. 10.12688/f1000research.24119.1 PMC7477648 32934805

[10] Blecharz-Klin K, Sznejder-Pachołek A, Wawer A, Pyrzanowska J, Piechal A, Joniec-Maciejak I, et al. Early exposure to paracetamol reduces level of testicular testosterone and changes gonadal expression of genes relevant for steroidogenesis in rats offspring. Drug Chem Toxicol. 2022;45(4):1862–9. 33657953 10.1080/01480545.2021.1892941

[11] Koehn LM, Huang Y, Habgood MD, Kysenius K, Crouch PJ, Dziegielewska KM, Saunders NR. Effects of paracetamol (acetaminophen) on gene expression and permeability properties of the rat placenta and fetal brain. F1000Res. 2020;9:573. 32934805 10.12688/f1000research.24119.1 PMC7477648

[12] Mehlisch DR. The efficacy of combination analgesic therapy in relieving dental pain. J Am Dent Association. 2002;133(7):861–71. 10.14219/jada.archive.2002.0300 12148679

[13] Rainsford KD. Ibuprofen: pharmacology, therapeutics and side effects. Springer; 2013.

[14] Volans G. Human toxicity of ibuprofen. In: Ibuprofen: discovery, development and therapeutics. 2015. pp. 498–517.

[15] Philibert L, Ngangue P, Lapierre J, Bernardino E, Kiki GM, Ntanda GM. Vulnerability analysis of Haitian adolescent girls before pregnancy: a qualitative study. Int J Adolesc Med Health. 2023;35(5):403–10. 37671939 10.1515/ijamh-2022-0114

[16] Fox CW, Zhang L, Moeller BC, Garzo VG, Chang RJ, Duleba AJ. Ibuprofen inhibits key genes involved in androgen production in theca–interstitial cells. F&S Sci. 2021;2(3):230–6. 35199048 10.1016/j.xfss.2021.06.004 PMC8862173

[17] Pemmari A, Paukkeri EL, Hämäläinen M, Leppänen T, Korhonen R, Moilanen E. MKP-1 promotes anti-inflammatory M (IL-4/IL-13) macrophage phenotype and mediates the anti-inflammatory effects of glucocorticoids. Basic Clin Pharmacol Toxicol. 2019;124(4):404–15. 30388313 10.1111/bcpt.13163

[18] Sabouri-Rad S, Hassanipour S, Hasankhani B. Evaluation of oxidative stress markers in paracetamol-induced hepatotoxicity: experimental and clinical evidence. Toxicol Rep. 2021;8:1324–33.34258234

[19] Zhu Y. Gap junction-dependent and-independent functions of Connexin43 in biology. Biology. 2022;11(2):283. 35205149 10.3390/biology11020283 PMC8869330

[20] Jang DI, Lee AH, Shin HY, Song HR, Park JH, Kang TB, et al. The role of tumor necrosis factor alpha (TNF-α) in autoimmune disease and current TNF-α inhibitors in therapeutics. Int J Mol Sci. 2021;22(5):2719. 33800290 10.3390/ijms22052719 PMC7962638

[21] M Lucero C, Marambio-Ruiz L, Balmazabal J, Prieto-Villalobos J, León M, Fernández P, I Gómez G. TNF-α plus IL-1β induces opposite regulation of Cx43 hemichannels and gap junctions in mesangial cells through a RhoA/ROCK-dependent pathway. Int J Mol Sci. 2022;23(17):10097. 36077498 10.3390/ijms231710097 PMC9456118

[22] Eugenín EA, Brañes MC, Berman JW, Sáez JC. TNF-α plus IFN-γ induce connexin43 expression and formation of gap junctions between human monocytes/macrophages that enhance physiological responses. J Immunol. 2003;170(3):1320–8. 12538692 10.4049/jimmunol.170.3.1320

[23] Tang M, Fang J. TNF-α regulates apoptosis of human vascular smooth muscle cells through gap junctions. Mol Med Rep. 2017;15(3):1407–11. 28075455 10.3892/mmr.2017.6106

[24] Decrock E, Vinken M, De Vuyst E, Krysko DV, D’herde K, Vanhaecke T, et al. Connexin-related signaling in cell death: to live or let die? Cell Death Differ. 2009;16(4):524–36. 19197295 10.1038/cdd.2008.196

[25] Hussein LM, Badawy MA, Gad SS, Abd EL. The prospective impact of paracetamol medication on female Wistar rats’ reproductive health (biochemical, genotoxic, and histological analysis). Egypt J Chem. 2025;68(9):1–16.

[26] Aprioku JS, Nwidu LL, Amadi CN. Evaluation of toxicological profile of ibuprofen in Wistar albino rats. Am J Biomed Sci. 2014;6(1):32–40.

[27] lışıcı D, Yılmaz S, Goktas B. Toxic, genotoxic and teratogenic effects of ibuprofen and its derivatives. Curr Drug Targets. 2023;24(4):361–70. 36600619 10.2174/1389450124666230104160435

[28] Gomaa S. Adverse effects induced by diclofenac, ibuprofen, and paracetamol toxicity on immunological and biochemical parameters in Swiss albino mice after repeated doses for one month. J Basic Appl Zool. 2018;79:1–9.

[29] Tripathi R, Pancholi SS, Tripathi P. Genotoxicity of ibuprofen in mouse bone marrow cells *in vivo*. Drug Chem Toxicol. 2012;35(4):389–92. 22283434 10.3109/01480545.2011.630670

[30] Coelho AM, Queiroz IF, Lima WG, Talvani A, Perucci LO, de Souza O, M., Costa DC. Temporal analysis of paracetamol-induced hepatotoxicity. Drug Chem Toxicol. 2023;46(3):472–81. 35313777 10.1080/01480545.2022.2052891

[31] American Veterinary Medical Association. AVMA Guidelines for the Euthanasia of Animals: 2020 ed. AVMA; 2020.

[32] Botrous S, Elmaghraby A, El-Achy S, Mustafa Y, Badr E, Haggag A, Abdel-Rahman S. Inhibition of TNF-α oncogene expression by Artemisia annua L. extract against pioglitazone side effects in male albino mice. Mol Biotechnol. 2024;66(3):432–41. 37179253 10.1007/s12033-023-00762-7 PMC10881748

[33] Shkedif HA, Abdelmonsif DA. Differentiation of bone marrow-derived mesenchymal stem cells into cardiomyocytes using different regimens of 5-azacytidine. Egypt J Histol. 2020;43(2):569–584.

[34] Botrous S, Elmaghraby A, Achy SE, et al. Prophylactic role of artemisinin in modulating FGFR3, HRAS, and TP53 to prevent early-stage urothelial carcinoma in BBN-induced mouse models. BMC Biotechnol. 2025;25:102. 10.1186/s12896-025-01039-4 10.1186/s12896-025-01039-4 PMC12442291 40963137

[35] Katturajan R, Vijayalakshmi S, Rasool M, Prince SE. Molecular toxicity of methotrexate in rheumatoid arthritis treatment: a novel perspective and therapeutic implications. Toxicol. 2021;461:152909. 10.1016/j.tox.2021.152909 34453959

[36] Livak KJ, Schmittgen TD. Analysis of relative gene expression data using real-time quantitative PCR and the 2 – ∆∆CT method. Methods. 2001;25(4):402–8. 11846609 10.1006/meth.2001.1262

[37] Eljaafari A, Pestel J, Le Magueresse-Battistoni B, Chanon S, Watson J, Robert M, et al. Adipose-tissue-derived mesenchymal stem cells mediate PD-L1 overexpression in the white adipose tissue of obese individuals, resulting in T cell dysfunction. Cells. 2021;10(10):2645. 34685625 10.3390/cells10102645 PMC8534339

[38] Capelo MF, Monteiro PB, Anastácio BM. Effects of major analgesics on male fertility: a systematic literature review. JBRA Assist Reprod. 2024;28(2):331. 38546117 10.5935/1518-0557.20240020 PMC11152418

[39] Gomaa S. Immunomodulatory and hematological effects induced by diclofenac, ibuprofen or paracetamol toxicity in Swiss albino mice. Eur J Biol Res. 2017;7(4):1–9.

[40] Rotundo L, Pyrsopoulos N. Liver injury induced by paracetamol and challenges associated with intentional and unintentional use. World J Hepatol. 2020;12(4):125. 32685105 10.4254/wjh.v12.i4.125 PMC7336293

[41] Aprioku JS, Nwidu LL, Amadi CN. Evaluation of toxicological profile of ibuprofen in Wistar albino rats. Am J Biomed Sci. 2014;6(1):32–40.

[42] Jones AF, Vale JA. Paracetamol poisoning and the kidney. J Clin Pharm Ther. 1993;18(1):5–8. 8473360 10.1111/j.1365-2710.1993.tb00560.x

[43] El-Mashad AER, El-Mahdy H, El Amrousy D, Elgendy M. Comparative study of the efficacy and safety of paracetamol, ibuprofen, and indomethacin in closure of patent ductus arteriosus in preterm neonates. Eur J Pediatr. 2017;176(2):233–40. 28004188 10.1007/s00431-016-2830-7

[44] Oyedeji KO, Bolarinwa AF, Akinbode AA. Effect of Corchorus olitorius extract on reproductive functions in male albino rats. Int J Pharm Pharm Sci. 2013;5(3):427–31.

[45] Wang Q, Xin B, Wang X, Li F, Fu H, Yan Z, Zhu Y. TT-10 may attenuate ibuprofen-induced ovarian injury in mice by activating COX2–PGE₂ and inhibiting Hippo pathway. Biomed Pharmacother. 2023;164:114981. 10.1016/j.biopha.2023.114981. 37984603 10.1016/j.reprotox.2023.108499

[46] Saad M, Flament J. Paracetamol overdose causing acute kidney injury without hepatotoxicity: a case report. Int J Emerg Med. 2024;17(1):81. 38956487 10.1186/s12245-024-00662-w PMC11220941

[47] Lanza FL, Collaku A, Liu DJ. Endoscopic comparison of gastroduodenal injury with over-the-counter doses of new fast-dissolving ibuprofen and paracetamol formulations: a randomized, placebo-controlled, 4-way crossover clinical trial. Clin Exp Gastroenterol. 2018;11:169–77. 29713191 10.2147/CEG.S153231 PMC5907787

[48] Sohail R, Mathew M, Patel KK, Reddy SA, Haider Z, Naria M, et al. Effects of non-steroidal anti-inflammatory drugs (NSAIDs) and gastroprotective NSAIDs on the gastrointestinal tract: a narrative review. Cureus. 2023;15(4):e37000. 37153279 10.7759/cureus.37080 PMC10156439

[49] Ho KY, Cardosa MS, Chaiamnuay S, Hidayat R, Ho HQT, Kamil O, et al. Practice advisory on the appropriate use of NSAIDs in primary care. J Pain Res. 2020;13:1925–39. 32821151 10.2147/JPR.S247781 PMC7422842

[50] Katturajan R, Prince SE. A role of connexin 43 on the drug-induced liver, kidney, and gastrointestinal tract toxicity with associated signaling pathways. Life Sci. 2021;280:119629.Tepedelen BE. Modulation of inflammatory mediators via ibuprofen in TNF-α treated benign prostatic hyperplasia cells. Mol Biol Rep. 2021;48(7):5747–5755. 10.1016/j.lfs.2021.119629 10.1016/j.lfs.2021.119629 34004253

[51] Cazanave S, Vadrot N, Tinel M, Berson A, Letteron P, Larosche I, Descatoire V, Feldmann G, Robin MA, Pessayre D. Ibuprofen administration attenuates serum TNF‑α levels, hepatic glutathione depletion, hepatic apoptosis and mouse mortality after Fas stimulation. Toxicology and Applied Pharmacology. 2008;231(3):336–343. 10.1016/j.taap.2008.05.010 10.1016/j.taap.2008.05.010 18572215

